# Tissue Inhibitor of Metalloproteinase–3 (TIMP-3) induces FAS dependent apoptosis in human vascular smooth muscle cells

**DOI:** 10.1371/journal.pone.0195116

**Published:** 2018-04-04

**Authors:** William R. English, Heather Ireland-Zecchini, Andrew H. Baker, Trevor D. Littlewood, Martin R. Bennett, Gillian Murphy

**Affiliations:** 1 Cancer Research UK Cambridge Research Institute, University of Cambridge, Li Ka Shing Centre, Robinson Way, Cambridge, United Kingdom; 2 Department of Oncology and Metabolism, University of Sheffield School of Medicine, Sheffield, United Kingdom; 3 Microscopy Core Facility, Cancer Research UK Cambridge Research Institute, University of Cambridge, Li Ka Shing Centre, Robinson Way, Cambridge, United Kingdom; 4 BHF Glasgow Cardiovascular Research Centre, Institute of Cardiovascular and Medical Sciences, University of Glasgow, Glasgow, United Kingdom; 5 University of Cambridge, Department of Biochemistry, Cambridge, United Kingdom; 6 Division of Cardiovascular Medicine, University of Cambridge, Addenbrooke's Hospital, Cambridge, United Kingdom; Qatar University College of Health Sciences, QATAR

## Abstract

Over expression of Tissue Inhibitor of Metalloproteinases-3 (TIMP-3) in vascular smooth muscle cells (VSMCs) induces apoptosis and reduces neointima formation occurring after saphenous vein interposition grafting or coronary stenting. In studies to address the mechanism of TIMP-3-driven apoptosis in human VSMCs we find that TIMP-3 increased activation of caspase-8 and apoptosis was inhibited by expression of Cytokine response modifier A (CrmA) and dominant negative FAS-Associated protein with Death Domain (FADD). TIMP-3 induced apoptosis did not cause mitochondrial depolarisation, increase activation of caspase-9 and was not inhibited by over-expression of B-cell Lymphoma 2 (Bcl2), indicating a mitochondrial independent/type-I death receptor pathway. TIMP-3 increased levels of the First Apoptosis Signal receptor (FAS) and depletion of FAS with shRNA showed TIMP-3-induced apoptosis was FAS dependent. TIMP-3 induced formation of the Death-Inducing Signalling Complex (DISC), as detected by immunoprecipitation and by immunofluorescence. Cellular-FADD-like IL-1 converting enzyme-Like Inhibitory Protein (c-FLIP) localised with FAS at the cell periphery in the absence of TIMP-3 and this localisation was lost on TIMP-3 expression with c-FLIP adopting a perinuclear localisation. Although TIMP-3 inhibited FAS shedding, this did not increase total surface levels of FAS but instead increased FAS levels within localised regions at the cell surface. A Disintegrin And Metalloproteinase 17 (ADAM17) is inhibited by TIMP-3 and depletion of ADAM17 with shRNA significantly decreased FAS shedding. However ADAM17 depletion did not induce apoptosis or replicate the effects of TIMP-3 by increasing localised clustering of cell surface FAS. ADAM17-depleted cells could activate caspase-3 when expressing levels of TIMP-3 that were otherwise sub-apoptotic, suggesting a partial role for ADAM17 mediated ectodomain shedding in TIMP-3 mediated apoptosis. We conclude that TIMP-3 induced apoptosis in VSMCs is highly dependent on FAS and is associated with changes in FAS and c-FLIP localisation, but is not solely dependent on shedding of the FAS ectodomain.

## Introduction

The mechanisms that regulate vascular smooth muscle cell (VSMC) proliferation, migration and extra-cellular matrix (ECM) invasion are key to understanding their role in the development of vascular pathology including atherosclerosis, neointima formation and restenosis [1, 2]. The Matrix Metalloproteinases (MMPs) are required for the degradation of the extracellular matrix (ECM) and more recently have been shown to process a wide range of proteins that regulate cell migration and proliferation [2–4]. A large number of studies demonstrate that MMP inhibition can prevent neointima formation, either with small peptide analogue inhibitors, or with the physiological inhibitors of MMPs, the Tissue Inhibitors of Metalloproteinases (TIMPs). However, low molecular weight broad-spectrum MMP inhibitors have shown variable ability to inhibit restenosis and intimal thickening, with ‘catch-up’ in neointima formation seen in some instances, or have proven ineffective [5]. Equally, not all of the TIMPs are effective in reducing neointima formation [6]. Of the TIMP studies, only TIMP-3 significantly reduced neointima formation in *in vivo* studies of saphenous vein/carotid artery interposition grafting or stenting and in *in vitro* human organ culture [6, 7]. TIMP-3 also decreases tumour growth in xenograft models indicating its potential as a therapeutic [8–11]. TIMP-3’s effectiveness is likely due to its combined ability to inhibit metalloproteinases (MPs), cause apoptosis and decrease proliferation in VSMCs [12, 13] and a variety of tumour-derived cell lines [9, 10, 14, 15]. Characterisation of the mechanism of TIMP-3-driven apoptosis in rat VSMCs and HeLa cells has shown that it is dependent on the TIMP-3 N-terminal MP active site binding ridge having the correct conformation, as apoptosis is lost on mutation of Cysteine-1 to Serine [16]. TIMP-3 induces apoptosis via a type-II death receptor pathway in rat VSMCs and HeLa cells. The type II pathway is death receptor dependent, yet subsequent caspase-8 activation cannot efficiently activate effector caspases. This is achieved through caspase-8 activation of the mitochondrial, intrinsic pathway and caspase-9 activation. Inhibition of TIMP-3 induced apoptosis with adenoviral expression of dominant-negative (DN) FAS-Associated protein with Death Domain (FADD), Bcl2 or Cytokine response modifier A (CrmA) blocked caspase-8 cleavage, subsequent mitochondrial depolarisation and caspase-9 activation in these cells [13]. Studies in melanoma and colon carcinoma cell lines have shown that a variety of death receptors are stabilised at the cell surface by adenoviral over-expression of TIMP-3, which increases sensitivity to death ligands [9, 17]. Here we show that TIMP-3-induced apoptosis in human VSMCs (hVSMCs) is via a type-I death receptor pathway that is highly dependent on FAS (CD95/Apo-1/TNFRSf6). The type-I pathway is able to activate effector caspases directly, without dependence on activation of the microchondrial/caspase-9 dependent amplification step. TIMP-3 increases FAS levels, induces the formation of the death-inducing signalling complex (DISC) and also regulates cellular-FADD-Like IL-1 converting enzyme-Like Inhibitory Protein (c-FLIP localisation). TIMP-3 also increases FAS at discrete cell surface regions, but does not increase overall surface FAS levels in hVSMCs. However, depletion of A Disintegrin And Metalloproteinase 17 (ADAM17), which is potently inhibited by TIMP-3 [18, 19] and is required for FAS shedding, does not replicate the apoptotic effects of TIMP-3, implicating ADAM17-independent effects of TIMP-3 on cellular apoptosis.

## Materials and methods

### Isolation and culture of human VSMCs

Human aortic vascular smooth muscle cells (hVSMCs) were isolated as described previously [20] or purchased from PromoCell GmbH (Heidelberg, Germany). VSMCs were cultured on plastic in DMEM, 10% v/v heat-inactivated supplemented bovine serum (SBS, Hyclone), glutamine, penicillin, streptomycin at 37°C and 5% CO_2_. Cells were passaged with trypsin/EDTA to a maximum of passage 8. All experiments were performed with cells at passage 6–8. All experiments were conducted in duplicate unless otherwise indicated and were consistent with a least two separate hVSMC isolates.

### Preparation and titration of recombinant adenovirus (RAd)

E1-deficient adenovirus expressing human TIMP-1, -2, -3 (RAd-T1, -T2 and T3), Cys1-Ser TIMP-3 (RAd-C1ST3), DN-FADD, CrmA, Bcl2, and containing the CMV promoter alone (RAd60), were prepared as described previously [13, 15]. RAd-EGFP was prepared by subcloning EGFP into pDC515 (Microbix) using standard PCR-based methods, checked by sequencing and then co-transfecting pBHGfrtΔE1E3 and 3FLP into NautCell™ 293s (Microbix). RAd-EGFP was then cloned as described previously [21]. Virus was titred against 293 cells (Microbix) by end-point dilution [21], Bicinchoninic Acid (BCA) assay and tested for replication-competent particles by serial dilution against HeLa cells obtained from ECACC.

### Adenoviral transduction of hVSMCs

hVSMC were seeded at 60–80% confluency. 24 h before infection the medium was replaced with growth medium containing 10% SBS. The minimum multiplicity of infection (MOI) required to obtain > 80% transduction was determined by infection with RAd-EGFP (determined as 100 pfu cell^-1^). If the cells were to be infected with more than one adenovirus, cells were infected for 2 h with up to 300 pfu cell^-1^ followed by 2 h in fresh medium before infection with 600 pfu cell^-1^ RAd-TIMP-3 for a further 2 h and a final medium change as described previously [13].

### Lentiviral transduction of hVSMCs

Lentiviral transduction of hVSMCs was chosen as a method for RNAi expression for target gene depletion as the time course of our experiments was too long for siRNA depletion and initial experiments using adenoviral transduction for expression of shRNA were inefficient. Lentiviral transduction did not alter the sensitivity of hVSMCs to TIMP-3 driven apoptosis. Lentivirus expressing shRNA from the U6 promoter, the puromycin resistance control virus PLKO, and non-targeting shRNA control virus were purchased from the Sigma Mission TRC shRNA library. Cells were transduced at 80% confluence in T25 flasks with magnetic-assisted transduction using the ExpressMag^TM^ lentiviral transduction kit according to the manufacturer’s instructions (Sigma Aldrich, UK). Cells were transduced at 1 pfu cell^-1^ and cultured for 48 h before passaging into medium with 1.2 μg ml^-1^ puromycin for 48 h. After puromycin selection, cells were starved of puromycin for 48 h before analysis by western blot or infection with adenovirus.

### Fluorogenic caspase-3 assay

After the appropriate treatments/infections, cells were harvested by scraping into lysis buffer after washing with PBS then centrifuged at 16,000 x g for 5 min to remove debris. 10 μl was dispensed into a black 96 well microtitre plate in quadruplicate for each sample. Samples were split into duplicate wells for each biological replicate and were incubated with caspase-3 assay buffer (50 mM Tris-HCl, pH 7.4, 5 mM EDTA, 1 mM DTT, 1 μM caspase-3 fluorogenic substrate IX (Merck) and Complete™ proteinase inhibitor cocktail (Roche) with and without 1 μM Z-VAD-CHO (Caspase Inhibitor II, Merck) at 37°C for 16 h. Fluorescence was measured in a TECAN Spectrafluor Plus microtitre plate reader at 485 nm and 595 nm. Caspase-3 activity was determined by subtraction of the Z-VAD-CHO-inhibited sample fluorescence from its paired sample without the caspase inhibitor.

### Analysis of nuclear fragmentation in hVSMCs by DAPI staining

hVSMCs were infected with 600 pfu/cell RAd-60, RAd-(C1S)T3, RAd-T3 for between 16 and 72 h. Cells were washed in HBSS with Mg^2+^ and Ca^2+^ (Invitrogen, UK) before fixing for 20 min with 4% w/v PFA in HBSS and mounting in Prolong Gold (Invitrogen, UK) mounting medium containing DAPI. DNA fragmentation was measured by counting nuclei that showed fragmentation/abnormal morphology and expressed as a percentage of the total nuclear count.

### Induction of apoptosis with staurosporine

To induce mitochondrial-dependent apoptosis, hVSMCs were infected with RAd60 or RAd-Bcl2 for 48 h before incubation with vehicle control (DMSO) or 0.1 μM staurosporine (Sigma) for 24 h. Lysates were harvested and caspase-3 activity measured as described previously.

### Measurement of total cellular mitochondrial membrane potential

To study differences in total cellular mitochondrial membrane potential, cells were infected with RAd60 or RAd-T3 for 48 h before incubation with 0.5 μM Tetramethyl Rhodamine Ethyl Ester (TMRE) and 1 μM Mitotracker Green (Invitrogen) in DMEM, 10% FCS, 37°C for 30 min. As a positive control for mitochondrial depolarisation, RAd60-infected cells were incubated with 5 μM Carbonyl cyanide-p-trifluoromethoxyphenylhydrazone (FCCP, Sigma) for 30 min before addition of mitochondrial dyes. Cells were removed from the culture surface by trypsinisation before re-suspension in PBS, 1% v/v paraformaldehyde and analysed by on a BD FACSCalibur flow cytometer.

### Cell lysis and western blotting

Cells were lysed in 1% v/v NP-40, 50 mM Tris-HCl, pH 7.4, 150 mM NaCl, 20 mM EDTA with Complete™ EDTA-free cocktail of proteinase inhibitors (Roche Diagnostics GmbH) and phosphatase inhibitor cocktail II (Invitrogen). Protein concentration was measured using the BCA method (Pierce, Thermo Scientific, UK) and samples adjusted so that equal protein concentrations were loaded in each well. After boiling in reducing Laemlli buffer for 5 min, proteins were resolved on 8 to 15% SDS-PAGE gels and transferred to nitrocellulose. The membrane was blocked in 5% fat-free milk in Tris-Buffered Saline, 0.05% v/v Tween-20 (TBS-Tween) for 30 min. Primary antibodies were used at the manufacturers’ recommended dilutions before washing in TBS-Tween and incubation with the appropriate secondary Horse Radish Peroxidase (HRP)-conjugated antibody before washing and visualisation by ECL. If the membrane was to be reprobed, membranes were treated with ReBlot-Strong^TM^ (Calbiochem) according to the manufacturer’s instructions. Antibodies were sourced as follows: Bcl2, Caspase-9 and cleaved PARP antibodies were from Cell Signalling Technologies (NEB, UK). Anti-FADD, c-FLIP and FAS Ligand (FASL) antibodies were from R & D Systems Europe (Oxford, UK). Anti-FAS (C-20) and anti-Caspase-8 antibodies were from SantaCruz (C20, S19). The anti-FAK antibody from was from BD-Biosciences. The mouse monoclonal antibody to human TIMP-3 was a kind gift from Fuji Biochemicals, Japan.

### Measurement of soluble FAS by ELISA

Soluble FAS (sFAS) was measured in the conditioned medium of hVSMCs using a human APO-1/FAS ELISA kit (BenderMed Systems). Cells were seeded in 6 well plates as described previously and sFAS measured according to the manufacturer’s instructions. sFAS was normalised with respect to cell number and is described as pg/ml FAS 10^5^ cells^-1^.

### Immunoprecipitation of DISC

hVSMCs were infected with either RAd60 or RAd-T3 at 600 pfu cell^-1^ for 48 h, washed in cold PBS and lysed by scraping into 250 μl Immuno-Precipitation (IP) buffer (20 mM Tris-HCl pH 7.4, 150 mM NaCl, 10 mM EDTA, 10% v/v glycerol, 1% v/v NP40, 1% v/v Triton-X100, 2 x Complete^TM^ proteinase inhibitor cocktail (Roche GmbH) and phosphatase inhibitor cocktail II (Invitrogen). Lysates were centrifuged at 6000 x g for 5 min to remove remaining debris and split into 50 μl aliquots which were diluted to 500 μl with ice cold IP buffer, and pre-cleared with 50 μl of a 50% v/v protein-G sepharose/IP buffer slurry (Sigma Aldrich, UK) for 1 h. After centrifugation at 300 x g for 5 min to remove the protein-G, immunoprecipitation (IP) was performed by addition of PBS, 2 μg FAS antibody (3D5, Alexis/Enzo Scientific) or mouse IgG control antibody (Jackson ImmunoResearch, Stratech, UK) and incubated with gentle agitation at 4°C for 2 h. 60 μl 50% v/v protein-G/IP buffer slurry was then added for 30 min at 4°C. The protein-G immuno-complexes were centrifuged at 300 x g for 5 min, washed three times with 500 μl ice cold IP buffer before boiling in Laemlli buffer and resolving on 10% SDS-PAGE. After transfer to nitrocellulose, blots were probed for caspase-8 (S-19) and FAS (C-20) and visualised by ECL.

### IFN-gamma sensitisation and induction of apoptosis with FASL-containing membrane vesicles

hVSMCs were stimulated with IFN-gamma (R & D Systems) at 10 ng ml^-1^ in PBS or a vehicle control for 24 h in low (1% v/v) serum media. An anti-FASL function blocking antibody (R & D Systems) or an isotype control antibody was then added at 2 μg ml^-1^ before membrane-bound FasL (1:500; Upstate Technology in DMEM) was added for a further 24 h before lysates were harvested and assayed for caspase activity as described previously [20].

### Flow cytometric analysis of surface FAS and FASL in hVSMCs

This was conducted as described by Rosner et al [20] for detection of FAS (Millipore, clone CH-11) and FASL, (R & D systems) using the appropriate immunoglobulin isotype controls and Alexafluor-488 secondary antibodies.

### Single cell image analysis of total surface FAS levels and FAS surface clustering in adherent hVSMCs

Cells were trypsinised 4 h after adenoviral transduction in 25 cm^2^ flasks and resuspended at a concentration of 2 x 10^4^ ml^-1^. For analysis of surface and total levels of FAS, hVSMCs were seeded in IbidiTreat μ-slides (Ibidi, Heidelberg, Germany). After adherence and spreading (24 h), the medium was changed and inhibitors added as required. The chambers were incubated for 72 h before washing with 3 x 120 μl Hanks-Buffered Saline Solution (HBSS) with Ca^2+^/Mg^2+^ and fixing in 120 μl PBS, 4% w/v paraformaldehyde (PFA) for 20 min. PFA was washed from the wells with 2 x 120 μl PBS followed by incubation for 5 min with 100 mM glycine in PBS. After washing once with PBS, cells were stained for FAS with 120 μl—mouse IgM monoclonal CH-11 (Millipore) or—isotype control (Sigma) at 5 μg μl^-1^ in PBS for 4 h. The wells were then washed with 3 x 120 μl PBS with 15 min incubation between each wash before incubating with Alexafluor-488 anti-mouse IgM (1:500, Invitrogen) in PBS for 2 h. Cells were labelled with 120 μl HCS CellMask^TM^ Deep Red dye (4 μM, Invitrogen) for 30 min. Wells were finally washed 3 x 120 μl for 15 min before imaging within 48 h on the iCys imaging cytometer (Compucyte). The cell periphery was selected using the CellMask^TM^ Deep Red fluorescence and expanding the contour generated by 2 pixels to ensure the true periphery was detected. Cell debris was removed from analysis by setting a minimum and maximum threshold for cell size and intensity. Total FAS antibody staining per cell was quantified by analysing Alexafluor-488 staining which only occurred within the cell periphery. To analyse the FAS spot-like surface staining, the threshold for staining intensity was increased and a minimal and maximal area threshold set for Alexafluor-488 staining that occurred within the cell periphery. Settings were established using the gallery function of the iCys imaging cytometer that allows images of the analysed events to be observed. These setting were maintained for all μ-slides within the same experiment. More detail on the methodology used for analysis of FAS staining can be found in Ireland-Zecchini et al [22].

### Immunofluorescence localisation of FAS within hVSMCs

hVSMCs were seeded onto 13 mm glass coverslips at 5 x 10^4^ ml^-1^ for 24 h before infection with control adenovirus (RAd60) RAd-T3 at 600 pfu cell^-1^ for 48 h. If cells were to be incubated with Choleratoxin B-Subunit (CTxB) AlexaFluor633 conjugates, these were added to the culture medium for 30 min. Coverslips were washed and fixed and blocked as described in the method for imaging cytometry before permeabilisation with 0.1% v/v Triton x-100 (Pierce, Thermofisher). Coverslips were then incubated with the following antibodies at 10 μg ml^-1^, for 1 h at room temperature; control mouse and rabbit immunoglobulins were from Jackson Immunochemicals (Chomapure IgG) or Sigma (Mouse IgM). Mouse IgM anti-human FAS antibody was from Millipore (CH-11), anti-C-FLIP, FADD and caspase-8 as described previously. Coverslips were washed 3 x 5 min in PBS before incubating with the appropriate secondary antibodies for 1 h: Alexafluor-488 and -633 anti-mouse IgM, Alexafluor-488 anti-mouse, anti-rabbit, anti-goat IgG and Alexafluor-547 anti-rabbit IgG (Invitrogen) and used at 1:500 dilution. The coverslips were then washed 3 x 5 min in PBS, MilliQ H_2_O, and mounted in ProlongGold containing DAPI (Invitrogen). For surface staining of FAS only, coverslips were stained with CH-11 without permeabilisation, washed and re-fixed for 10 min in 2% v/v PFA before permeabilising and incubation with Alexfluor-633-Phalloidin (Invitrogen) according to the manufacturer’s instructions.

### Statistical analysis

Data were analysed using Graph Pad Prism. Pair wise comparisons were made using Student’s *t*-Test. Where more than two parameters were analysed, ANOVA with a Tukey post test was used. Values of *n* are for biological replicates and each biological replicate is the mean of 3 technical replicates.

## Results

### TIMP-3-induced apoptosis in hVSMCs is via a type I pathway

**Fig 1:**
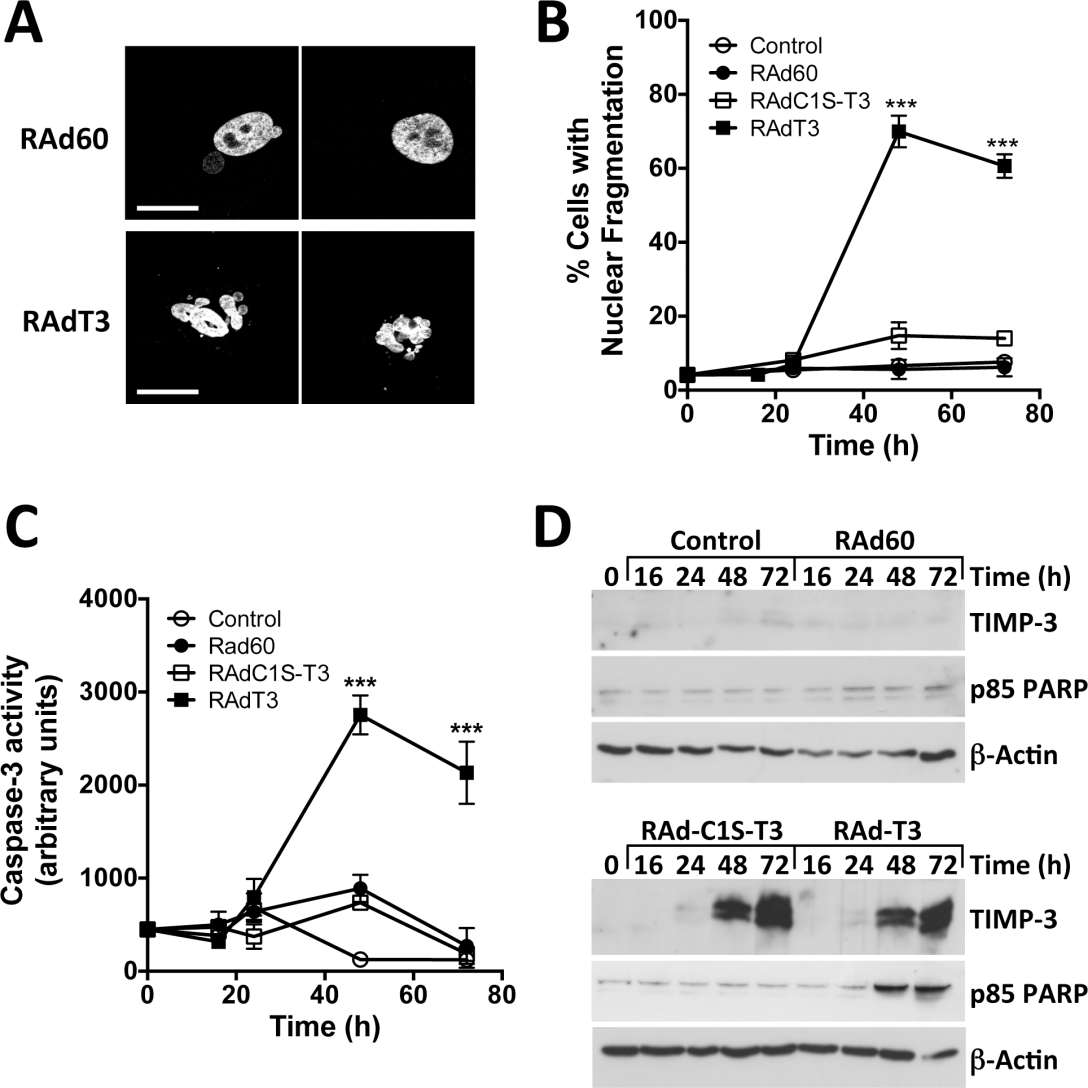
TIMP-3 overexpression in hVSMCs induces apoptosis. A and B. Nuclear fragmentation measured by DAPI staining (examples in A for RAd60 and RAdT3, scale bar = 20 μm) expressed as percentage of total nuclei count for VSMCs infected with control adenoviral particles (RAd60), TIMP-3 expressing adenovirus (RAdT3) or Cys1-Ser TIMP-3 (RAd-(C1S)T3). Data are mean ± SEM, n = 3. *** = P < 0.001. C. Caspase-3 activity measured in hVSMC lysates at different time points post infection without or with RAd60, RAdT3 or RAd-C1S-T3. Data are mean ± SEM, n = 3. *** = P < 0.001. D. Western blot of hVSMC lysates harvested at the indicated time points probed for the 85 kDa caspase-3 cleavage product of PARP, TIMP-3 and re-probed for beta-actin as a loading control.

**Fig 2:**
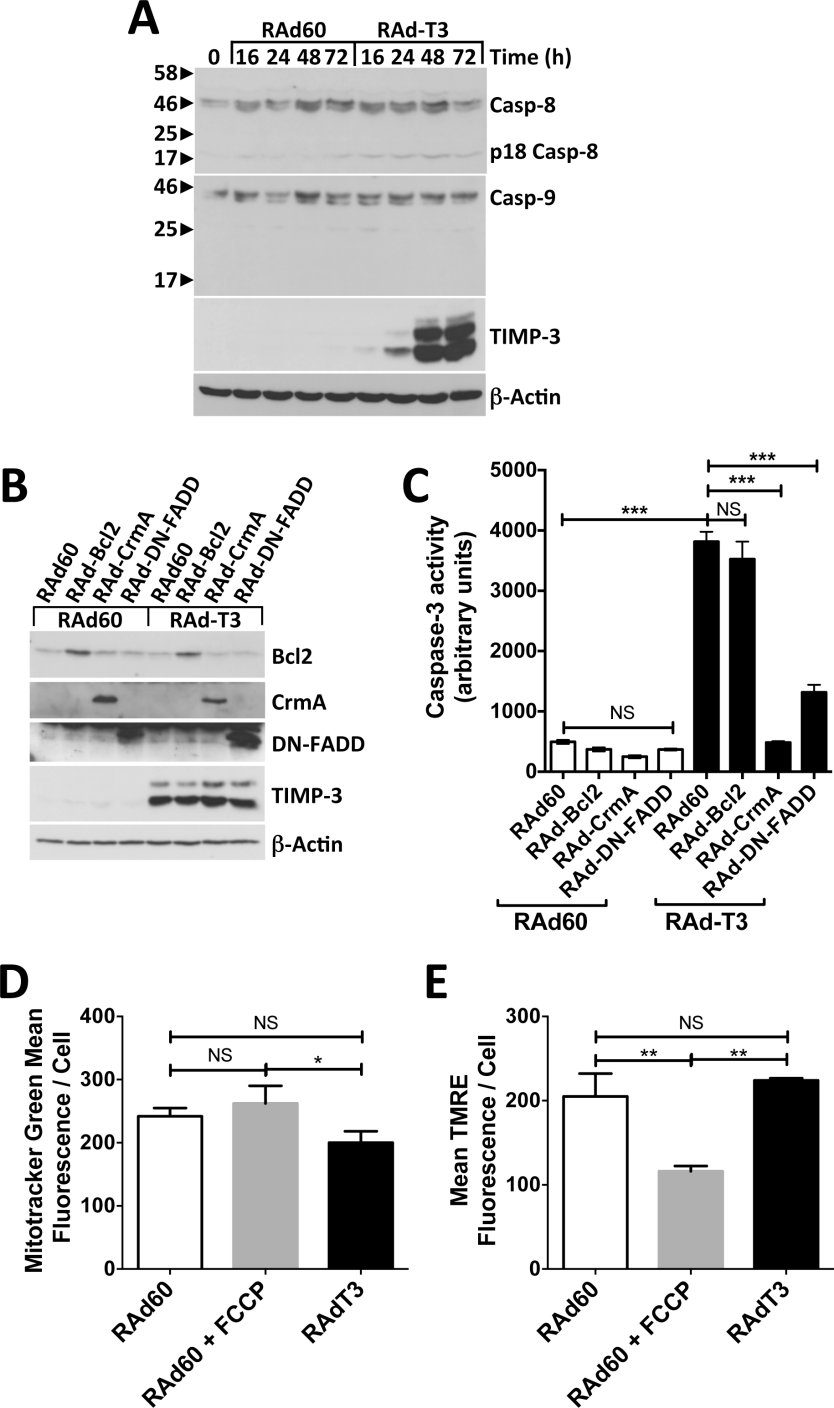
TIMP-3 overexpression induces apoptosis via a type-I death receptor pathway in hVSMCs. A. Western blot of lysates of VSMCs infected with either control (RAd60) or TIMP-3 (RAdT3)-expressing adenovirus harvested at the indicated times. Western blots were probed with anti-caspase-8, which can detect the pro, processed and 18 kDa active forms (Casp-8 and p18 Casp-8 respectively), anti-caspase-9 (Casp-9), anti-TIMP-3 and anti-beta-actin as a loading control. B and C. hVSMCs were infected with control (RAd60) or TIMP-3 (RAdT3)-expressing adenovirus followed by control (RAd60), Bcl2 (RAd-Bcl2), CrmA (RAd-CrmA) or dominant negative FADD (RAd-DN-FADD)-expressing adenovirus as described in the experimental procedures. Lysates were harvested after 72 h and analysed by Western blot to confirm expression of Bcl2, CrmA, DN-FADD and TIMP-3 with anti-beta-actin as a loading control (B) or caspase-3 activity measured (C). Data in C are the mean ± SEM, n = 5, *** = P < 0.001, NS = Not Significant. D and E. hVSMCs were infected with control (RAd60) or TIMP-3 (RAdT3)-expressing adenovirus for 48 h before incubation with both mitotracker green to measure total mitochondrial levels and TMRE to detect microchondrial depolarisation. RAd60 cells were also incubated with FCCP as a positive control for mictochondrial depolarisation. Mean fluorescence intensity ± SEM, n = 5 of mitotracker green (D) and TMRE (E) was measured using flowcytometry (example data shown in S1B Fig). * = P < 0.05, ** = P < 0.01, NS = Not Significant.

We first confirmed that TIMP-3 induced hVSMC apoptosis as previously described [13, 16]. After optimisation of the multiplicity of infection (MOI) to achieve greater than 80% transduction using RAd-EGFP (100 pfu cell^-1^), human VSMC apoptosis was induced by RAd-T3 at an MOI of 600, with maximal cell death observed 48–72 h after transduction as measured by detecting nuclear fragmentation on DAPI staining (Fig 1A and 1B). Control adenovirus (RAd60) or cells transduced with virus expressing C1S TIMP-3, which is unable to inhibit metalloproteinases (RAd-(C1S)T3 [16]) were ineffective. Maximal caspase-3 activity was detected between 48 and 72 h (Fig 1C) when cellular TIMP-3 protein levels had also reached significant levels. This also coincided with increased levels of the p85 caspase-3 cleavage fragment of PARP (Fig 1D). Western blotting for caspase-8 revealed that the p18 form was detectable at earlier times points after RAd-T3 than in RAd60-infected cells and increased over time, peaking at 48 h. Levels of caspase-9 remained unchanged and only very low levels could be detected (Fig 2A). To further characterise the mechanism of apoptosis we co-infected hVSMCs with adenovirus expressing dominant negative FADD (DN-FADD), CrmA (a viral caspase-8 inhibitor), or Bcl2, an inhibitor of mitochondrial depolarisation [13]. Apoptosis of hVSMCs was significantly inhibited by DN-FADD or CrmA, but not Bcl2 (Fig 2B and 2C). To confirm Bcl2 could inhibit apoptosis in hVSMCs in light of this negative result we found overexpression did inhibit staurosporine-induced apoptosis, known to activate mitochondrial dependent apoptosis, indicating Bcl2 expression levels were functional (S1A Fig). RAd-T3-infected cells did not show alterations in total levels of mitochondria or mitochondrial membrane potential as detected by uptake of the dyes Mitotracker Green and TMRE respectively, in contrast to FCCP, known to activate mitochondrial depolarisation, supporting the lack of involvement of a type-II pathway (S1B Fig and Fig 2D and 2E). Together, our data indicate that TIMP-3-induced apoptosis in hVSMCs is predominantly via a type-I death receptor pathway [23].

### TIMP-3-induced apoptosis is dependent on FAS, induces DISC formation and regulates c-FLIP localisation

**Fig 3:**
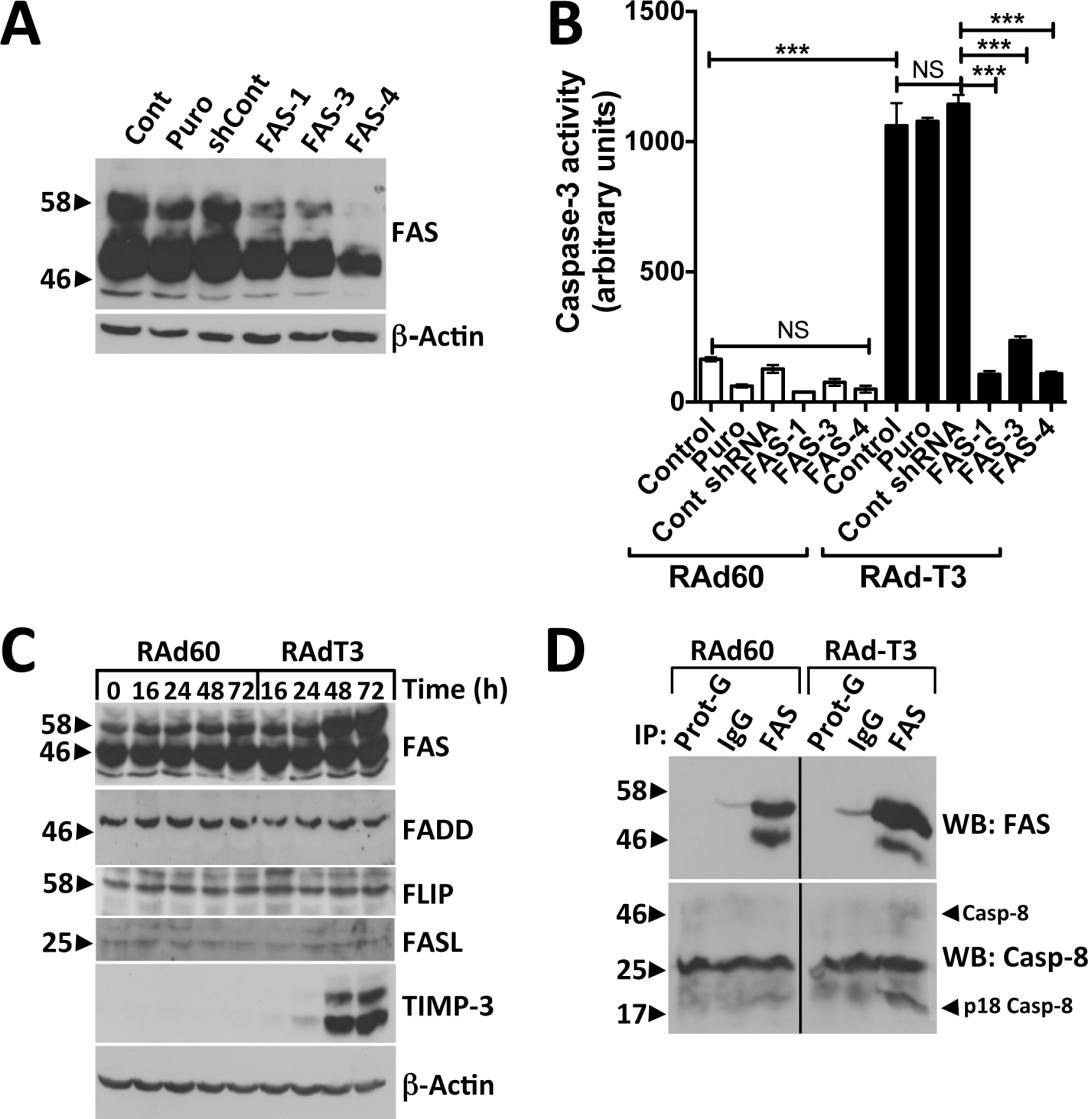
TIMP-3-induced apoptosis in hVSMCs is dependent on FAS, increases cellular FAS protein levels and induces DISC formation. A. Western blots of hVSMCs (Cont), transduced with lentivirus carrying puromycin resistance alone (Puro), expressing non-targeting control shRNA (shCont), or shRNA targeting FAS (FAS-1, -3, -4). B. Cells from (A) were infected with control (RAd60) or TIMP-3-expressing adenovirus (RAdT3) and caspase-3 activity analysed after 72 h. Data are the mean ± standard deviation, n = 3, *** = p < 0.001, NS = Not Significant. C. Western blots for FAS, FADD, FLIP, TIMP-3 and beta-actin in hVSMCs transduced with RAd60 or RAdT3 at the indicated time points. D. Western blots of VSMCs infected for 48 h with RAd60 or RAdT3 before immunoprecipitation of lysates with protein-G beads alone, IgG isotype control or anti-FAS monoclonal 3D5. Western blots were probed for caspase-8 then re-probed for FAS using anti-FAS IgM monoclonal CH-11. The band corresponding to procaspase-8 and the p18 form are indicated by Casp-8 and p18 Casp-8 respectively. A 26 kDa non-specific band was also detected. Empty lanes used to prevent carryover of the ECL signal on long exposure from RAd60 to RAdT3 immunoprecipitate containing lanes have been cropped from the image.

FAS is a death receptor that can induce type-I apoptotic pathways, and is highly expressed in hVSMCs [23, 24]. To demonstrate whether TIMP-3-induced apoptosis required FAS, hVSMCs were transduced with lentivirus expressing shRNAs targeting FAS. Three shRNA from the Sigma Mission TRC1 library reduced FAS protein levels significantly by western blot and by ELISA. Two shRNA reduced FAS in cell lysates by 50%, one by >60% (Fig 3A) and also led to a reduction of soluble FAS in the culture medium as detected by ELISA (S2 Fig). FAS depletion significantly reduced TIMP-3-induced caspase activation (Fig 3B). TIMP-3 overexpression increased protein levels of FAS but did not change total cellular levels of proteins that comprise or regulate the Death-Inducing Signalling Complex (DISC) including FADD, 55 kDa c-FLIP and FASL (Fig 3C). To identify whether TIMP-3 induces formation of DISC, FAS was immunoprecipitated using lysates from RAd60 or RAd-T3-infected cells, and immuno-complexes probed for caspase-8 by western blot. Increased levels of 50 and 18 kDa bands were detected at 48 h (Fig 3D) in TIMP-3 expressing cells compared to RAd60 control virus-infected cells, indicating increased association of pro- and active-caspase-8 with FAS. DISC formation was not detectable at 72 h (data not shown).

**Fig 4:**
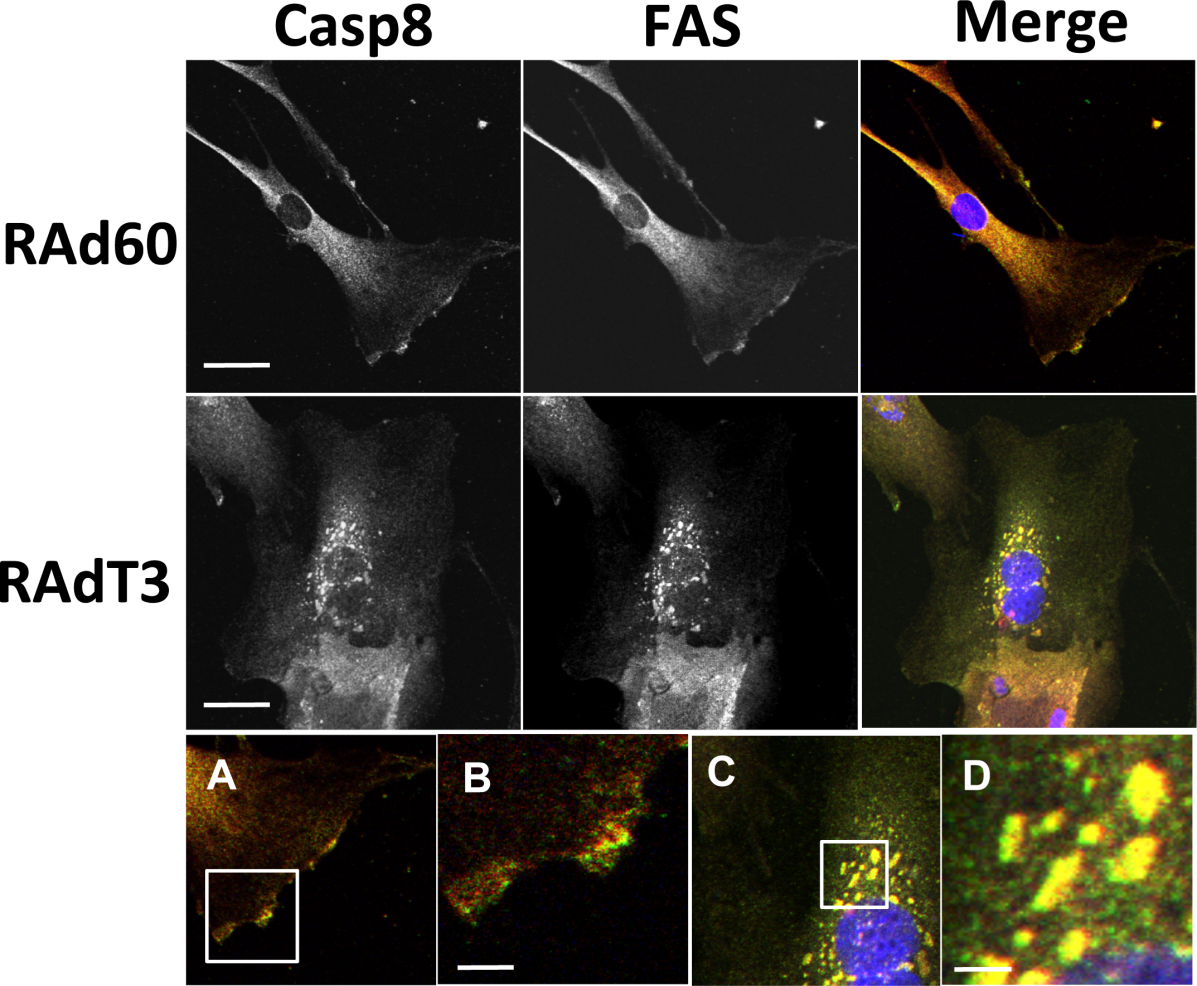
TIMP-3 induces a change in the cellular localisation of FAS and caspase-8 in hVSMCs. Immunocytochemistry for caspase-8 (red in overlay) and FAS (green in overlay) with nuclei in blue (DAPI) in the overlay images, in VSMCs infected with either control (RAd60) or RAdT3 adenovirus for 48 h. A and B are magnified images of RAd60-infected cells stained for caspase-8 and FAS shown in the upper panels. C and D are magnified images of RAdT3-infected cells stained for caspase-8 and FAS taken from the upper panels. Scale bars represent 36 μm in the upper panels and 7 μm in panels A to D.

**Fig 5:**
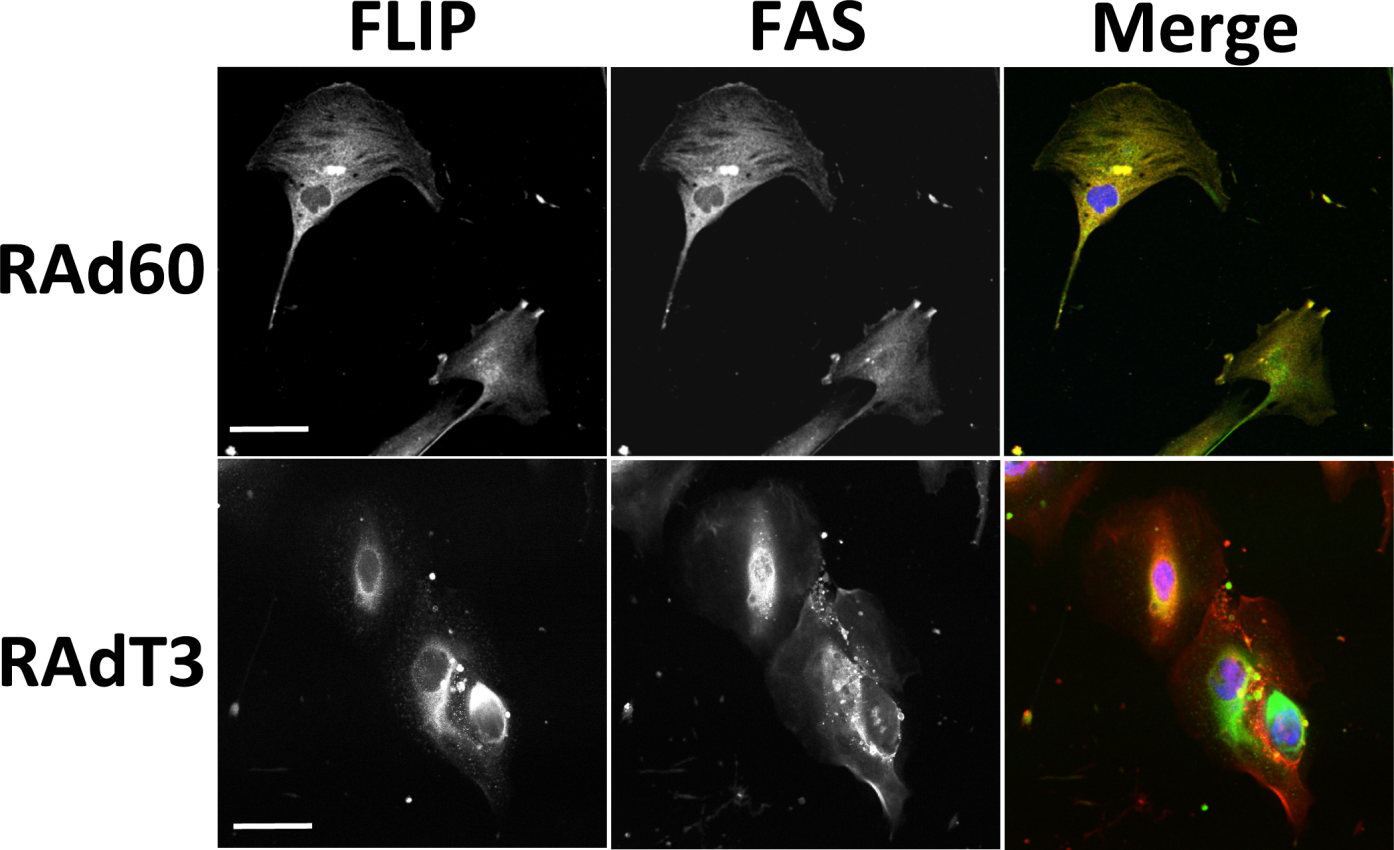
TIMP-3 induces the cellular redistribution of c-FLIP. Immunocytochemistry for c-FLIP and FAS in VSMCs infected with either control (RAd60) or RAdT3 adenovirus for 48 h. c-FLIP is shown in green, FAS in red and nuclei in blue (DAPI) in the overlay images. Scale bar represents 67 μm.

To further confirm DISC formation, and to explore the relationship between c-FLIP and FAS, the localisation of FAS, c-FLIP and caspase-8 was examined by immunofluorescence staining. In the absence of TIMP-3, FAS and caspase-8 were distributed throughout the cytosol and at the leading edge of cells, but showed poor co-localisation (Fig 4A and 4B). 48 h after RAd-T3 infection, the localisation of both proteins at the cells edge was lost and FAS and caspase-8 showed a strong intracellular localisation in vesicles (Fig 4C and 4D). A similar localisation pattern was observed for FADD and FAS (S3 Fig). FAS and c-FLIP localised near the leading edge of cells, and on the addition of RAd-T3, c-FLIP gained a strong perinuclear localisation and no longer localised with FAS (Fig 5). However we were unable to co-immunoprecipitate c-FLIP with FAS or caspase-8 indicating that they are either not in a directly interacting complex or the interaction is too weak to detect (data not shown). Activated FAS has also been shown to co-localise with the lipid raft marker Cholera toxin B-subunit that associates with G_M1_-lipids (CTxB, [25]). In RAd60-infected VSMCs, FAS and FADD localised with CTxB-stained regions at the cell’s leading edge and peri-nuclear vesicles, whereas in RAd-T3-infected cells, FAS, FADD and CTxB localised predominantly in intracellular vesicles (S3 Fig).

### TIMP-3-induced apoptosis is independent of FASL.

**Fig 6:**
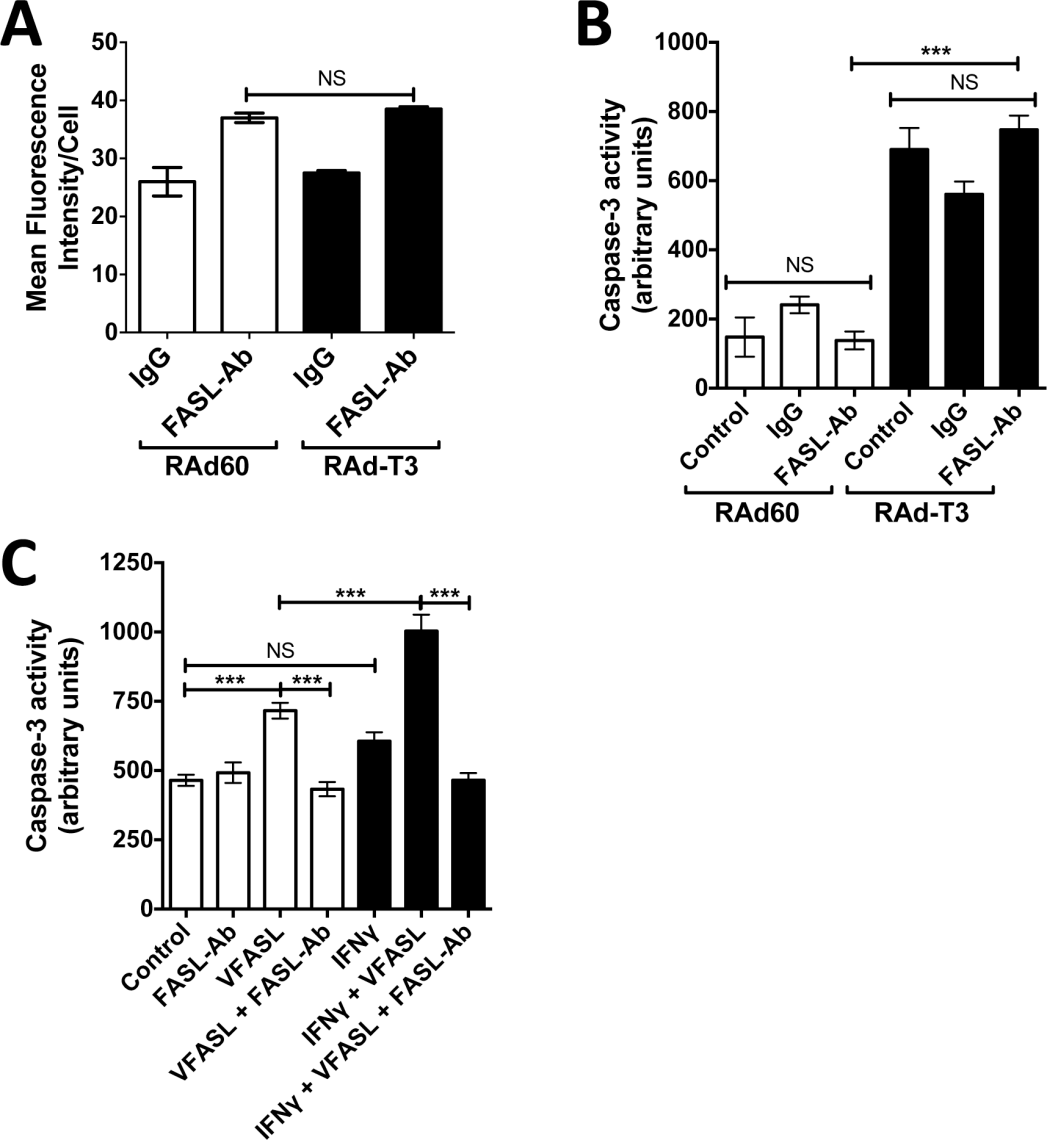
TIMP-3 induced apoptosis shows independence of FASL. A. hVSMCs were transduced with control adenovirus (RAd60) or adenovirus expressing TIMP-3 (RAdT3) for 48 h before staining with 2 μg ml^-1^ isotype control IgG or an anti-FASL antibody and an anti-IgG Alexfluor488 conjugate. Cell-surface alexafluor488 mean fluorescence staining intensity was measured using flowcytometry. Data is the mean ± SEM, n = 3. NS = Not Significant. B. hVSMCs were transduced with control adenovirus (RAd60) or adenovirus expressing TIMP-3 (RAdT3) for 16 h before the addition of PBS (vehicle control), 2 μg ml^-1^ isotype control IgG or a function blocking anti-FASL antibody. The antibody was added every 24 h before cell lysates were harvested 72 h post adenoviral transduction and caspase-3 activity measured. Data is the mean ± SEM, n = 3. *** = P < 0.001, NS = Not Significant. C. In order to demonstrate the function blocking FASL antibody could block FASL dependent apoptosis, hVSMCs were stimulated with either PBS vehicle control or 10 ng ml^-1^ IFN-gamma for 24 h before the addition of FASL in membrane vesicles (vFASL), 2 μg ml^-1^ isotype control IgG or a functional blocking FASL antibody. 24 h after the addition of antibody lysates were harvested and caspase-3 activity measured. Data are the mean ± SEM, n = 3. *** = P < 0.001, NS = Not Significant.

hVSMCs have been shown to express low levels of FASL, [24]. We also found that FASL is detectable at low levels at the cell surface by flow cytometry (Fig 6A), and the very low levels detected by western blot did not change on expression of TIMP-3 (Fig 3C). Furthermore, whilst an inhibitory antibody to FASL inhibited caspase-3 activation in IFN-gamma sensitised cells exposed to FASL in cell membrane vesicles [20], it did not inhibit TIMP-3 induced apoptosis (Fig 6B and 6C).

### TIMP-3 decreases FAS shedding and increases FAS at the surface of hVSMCs at discrete localisations

**Fig 7:**
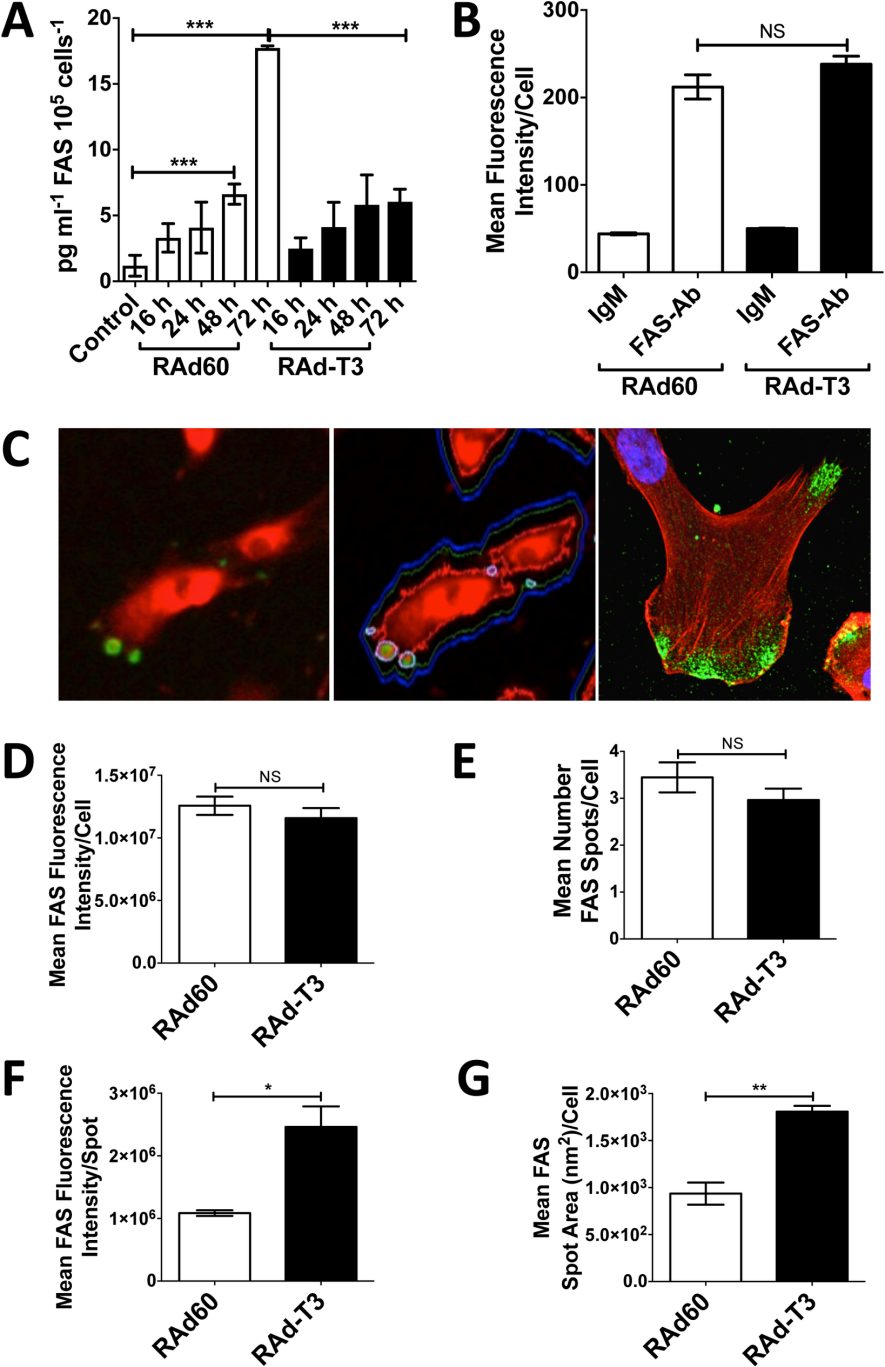
TIMP-3 increases FAS at the surface of hVSMCs at distinct regions. A. Soluble FAS (pg ml^-1^) in the culture media of hVSMCs transduced with control (RAd60) or TIMP-3 (RAdT3)-expressing adenovirus at the time points indicated. Data are the mean ± SEM, n = 3. *** = P < 0.001. B. Surface levels of FAS analysed after 48 h by flowcytometry in hVSMCs infected with RAd60 or RAdT3-expressing adenovirus. Cells were stained with an IgM isotype control antibody or with anti-FAS IgM (mAb CH-11) followed by an anti-IgM Alexfluor-488 secondary antibody. Data is the mean fluorescence intensity ± SEM, n = 3, NS = Not Significant. C. Left; example hVSMCs in μ-slides stained with CellMask^TM^ Deep Red dye and for surface FAS with mAb CH-11 and an anti IgM Alexafluor-488 secondary antibody acquired by the iCys system. Middle; Image analysis contours superimposed on the left panel image generated by the iCys system. Contours in green are at the detection limit for the cell tracker dye, contours in blue are an enlargement of the contour in green to enable detection of the true cell periphery. Contours in white are the focal FAS surface staining detected. Right; Confocal image of surface staining of FAS with mAb CH-11 and an anti IgM Alexafluor-488 secondary antibody and counterstained with DAPI and Phalloidin-Alexafluor633. D–G. Quantification of FAS surface staining per cell in μ-slides by image analysis with the iCys system with approximately 70–100 cells detected per well. D. Analysis of total FAS fluorescence within the cell periphery analogous to the flowcytometry experiment in B. E. Number of spot-like FAS positive structures at the cell surface. F. Analysis of the intensity of staining within the spot-like FAS positive structures. G. Analysis of the area of spot-like FAS positive structures. Data are mean ± SEM, n = 6. * = P < 0.05, ** = P < 0.01, NS = Not Significant.

As TIMP-3 inhibition of FAS ectodomain shedding could be the cause of the increase in cellular FAS, soluble FAS (sFAS) was measured in cell-conditioned medium by ELISA. TIMP-3 expression significantly reduced sFAS in the cell-conditioned medium (Fig 7A). Adenoviral over-expression of TIMP-1 or -2 did not inhibit FAS shedding or induce apoptosis (S4A and S4B Fig). 10 μM BB-94 or 50 μM GM6001, broad-spectrum MMP and ADAM inhibitors, were only able to weakly inhibit FAS shedding, and did not induce significant caspase-3 activation, even if added daily (S4A and S4B Fig). As flow cytometry did not detect a significant increase in total surface FAS levels at 72 h on TIMP-3 over-expression (Fig 7B), FAS cell surface localisation was further analysed using single cell image analysis of FAS staining in adherent hVSMCs. Microfluidic chamber slides (μ-slides) were used to ensure that cells were distributed evenly on the culture surface maximising the number of single cells not contacting neighbours, allowing quantification of FAS levels per cell [22]. By using cell membrane labelling dyes, the cell periphery could be identified allowing FAS levels to be quantified per cell after immunofluorescence labelling (Fig 7C). Measurement of total FAS surface staining intensity within the cell’s periphery, analogous to the flow cytometry data, showed no significant change in FAS levels in TIMP-3-overexpressing cells (Fig 7D). Total cellular FAS staining was significantly reduced using the shRNA characterised previously (S5 Fig), confirming our measurement of surface FAS using this method. FAS immunofluorescence staining was concentrated at discrete spot-like regions at the surface, typically close to the cell’s edge, where punctate staining of surface FAS was concentrated, (Fig 7C). The number of these spot-like regions did not change significantly per cell on overexpression of TIMP-3 (Fig 7E). However, TIMP-3 overexpression did significantly increase the intensity of FAS staining in these regions and also increased their area (Fig 7F and 7G).

### The role of ADAM17 in FAS shedding

**Fig 8:**
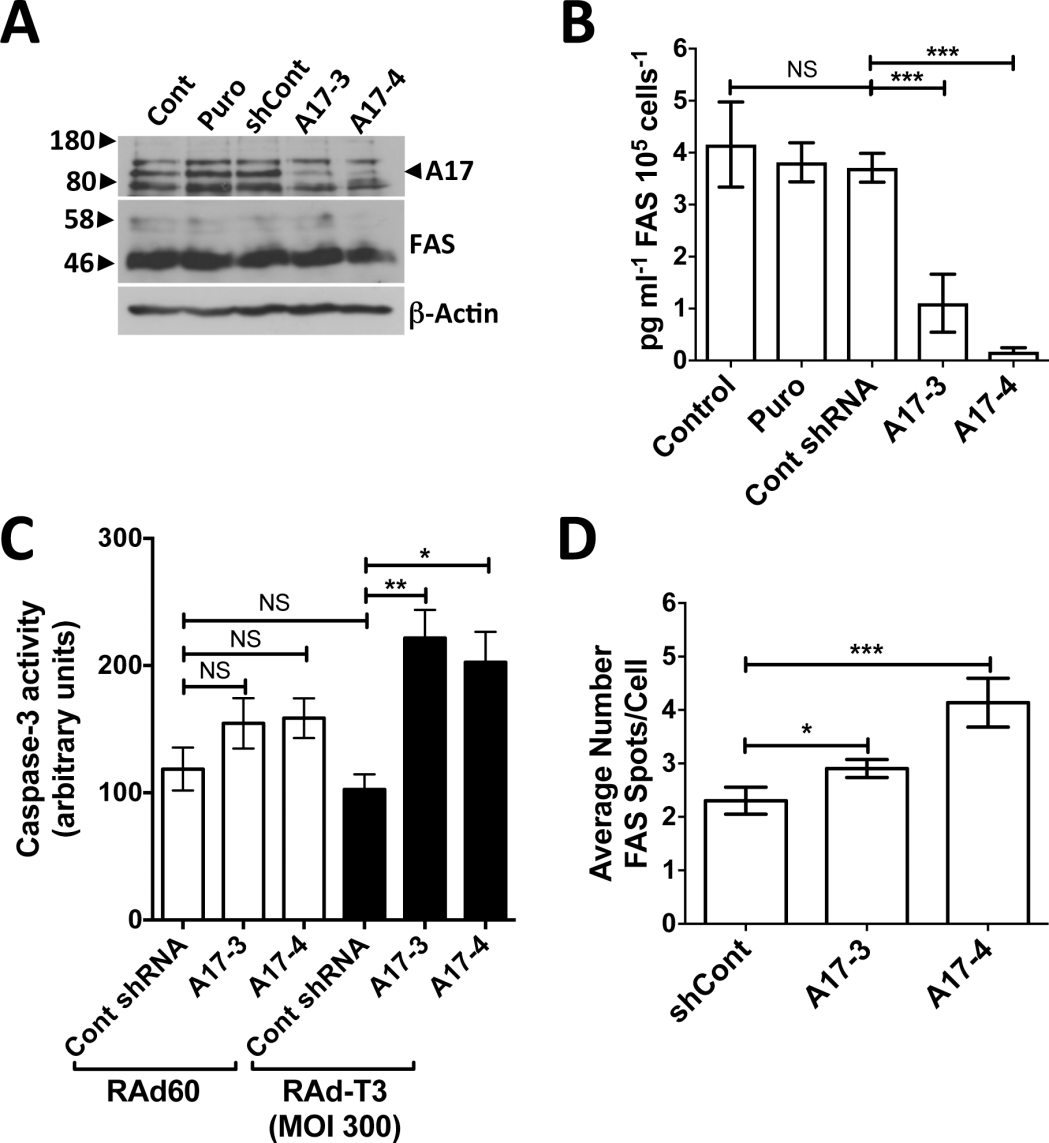
ADAM17 depletion in hVSMCs regulates FAS shedding and increases sensitisation to TIMP-3-dependent apoptosis. A. Western blot for ADAM17 (A17) or FAS in hVSMCs (Cont), infected with lentivirus conferring puromycin resistance alone (Puro), control non-targeting shRNA (shCont) or shRNA targeting ADAM17 (A17-3 and A17-4). ADAM17 is the middle band, flanked by non-specific bands. B. Soluble FAS levels measured by ELISA in cell medium after 72 h from cells in A. Data are means ± SEM, n = 3. *** = P < 0.001, NS = Not Significant. C. Caspase-3 activity in hVSMC lysates from cell transduced with lentivirus expressing control shRNA or ADAM17 targeting shRNA (A17-3 and A17-4) infected with 300 pfu cell^-1^ RAd60 or RAdT3-expressing adenovirus. Data are means ± SEM, n = 3. * = P < 0.05, ** = P < 0.01, NS = Not Significant. D. Quantification of surface FAS by single cell image analysis, data shows the mean number of spot-like FAS surface structures per cell, data are means ± SEM, n = 3. * = P < 0.05, *** = P < 0.001.

The metalloproteinase(s) involved in TIMP-3 mediated apoptosis have not been identified and the metalloproteinases that contribute to FAS shedding in hVSMCs are unknown. To investigate if ADAM17, a metalloproteinase which is inhibited by TIMP-3, but not TIMP-1 or -2, is involved in FAS shedding [18, 19], ADAM17 was depleted from hVSMCs using lentiviral shRNA transduction (Fig 8A). ADAM17 depletion did not increase total levels of FAS as detected by western-blot (Fig 8A). ADAM17 depletion did cause a decrease in FAS shed into the culture medium but did not increase caspase-3 activation to the same extent as TIMP-3 over-expression (Fig 8B and 8C). ADAM17 shRNA-expressing cells did show increased sensitivity to TIMP-3-induced apoptosis. Infection of hVSMCs with 300 pfu cell^-1^ RAd-T3 for 72 h did not increase caspase-3 activation over control virus-infected cells. However, the addition of 300 pfu cell^-1^ RAd-T3 to hVSMCs depleted of ADAM17 increased caspase-3 activation (Fig 8C). As TIMP-3 increased FAS at cell surface sub-localisations, we investigated if ADAM17 depletion also caused a similar increase. Quantification of surface staining of FAS in ADAM17 shRNA-expressing cells using single cell image analysis showed that, unlike cells overexpressing TIMP-3, FAS cell surface spot-like regions increased in number, but did not change significantly in measurements of area, intensity or circularity (Fig 8D and S6A and S6B Fig).

## Discussion

Current published data suggest that TIMP-3 induces apoptosis via a type-II death receptor mechanism in some cell types [13], although both caspase and death receptor independent mechanisms have also been proposed [26, 27]. However, although TIMP-3 overexpression shows potential as a therapeutic agent in human vascular disease [6], no studies have documented the mechanism of apoptosis in human vascular smooth muscle cells.

Our data show that TIMP-3 overexpression induces apoptosis with similar kinetics and via a caspase-dependent mechanism as described previously [8, 9, 12, 14, 15, 28]. We did not detect induction of senescence on expression of TIMP-3, but did find TIMP-3 was unable to induce cell death in senescent cells (unpublished observations). However, in contrast to studies in human cancer cell lines or rat VSMCs, TIMP-3 induced apoptosis in hVSMCs is via a FAS-dependent type-I apoptotic pathway, as Bcl2 over expression did not inhibit apoptosis significantly and loss of mitochondrial membrane potential was not seen [23]. TIMP-3-induced apoptosis in hVSMCs was found to be highly dependent on FAS, a prominent activator of type-I apoptotic pathways. FAS can be glycosylated with a molecular weight of approximately 45 to 55 kDa on SDS-PAGE, which is due to both N- and O-linked glycosylation. Increases in the 50–55 kDa glycosylated form of FAS has been shown to down-regulate sensitivity of T- and B-cells cells to ligand-induced apoptosis [29, 30]. TIMP-3 also increased the glycosylated 50–55 kDa form of FAS in hVSMCs, yet this appears to correlate with increased apoptosis, suggesting the relationship between FAS glycosylation and apoptosis may not be clearly defined and cell type-specific.

Mechanistic studies to date have shown that TIMP-3 overexpression inhibits the ectodomain shedding of TNFRSfs, leading to an accumulation at the cell surface, either to sufficient levels to induce apoptosis in the absence of TNFRSf ligands [9, 13], or to sufficient levels that cells are sensitised to ligand-induced apoptosis [17]. Although FAS was detectable at the cell surface of hVSMCs, TIMP-3 did not significantly increase the overall levels of surface FAS, despite the increase in total FAS levels detected by western blot, which also differs from the effects of TIMP-3 observed previously [9, 17]. This indicates that the regulation of FAS at the cell surface by TIMP-3 in hVSMCs differs from its regulation by IFN-gamma, which increases surface FAS levels yet does not induce apoptosis in the absence of FASL [20]. FAS levels did increase at localised surface regions, typically near the cell’s leading edge. As FAS levels increased in RAd-T3-infected cells and TIMP-3 has been implicated in the inhibition of ectodomain shedding of TNFRSf members, we also explored the role of FAS shedding. As previously shown, mutation of Cys1 to Ser, which destabilises the N-terminus of TIMPs and prevents their interaction with metalloproteinases, did not activate apoptosis, suggesting TIMP-3 exerts its apoptotic effect in hVSMCs at least in part through inhibition of metalloproteinases [16]. Although FAS has been shown to be shed by MMP-7 in tumour cells [31], and addition of recombinant MMP-7 to hVSMCs did increase levels of sFAS (WRE unpublished data), we were unable to inhibit sFAS production with adenoviral overexpression of TIMP-1 or -2 which are potent inhibitors of MMP-7 [32]. As shedding was only significantly inhibited by TIMP-3, we investigated whether ADAM17 is involved in FAS shedding, as it has been implicated in the shedding of other TNFRSf members [33, 34] and is potently inhibited by TIMP-3 and not TIMP-1 or TIMP-2 [18, 19]. Although ADAM17 depletion reduced sFAS significantly, this did not induce apoptosis or replicate the surface clustering or aggregation of FAS seen on TIMP-3 expression. This suggests that inhibition of FAS shedding may not be the main mechanism of induction of apoptosis by TIMP-3 in hVSMCs, and that inhibition of other proteinases may also be required, or that TIMP-3 functions in part independently of its role as metalloproteinase inhibitor.

Clustering and pre-aggregation of FAS at the cell plasma membrane has been proposed to be a key prerequisite prior to endocytosis and efficient apoptosis via DISC formation in intracellular compartments in type-I cells [23]. Previous studies have shown FAS to be localised in intracellular compartments such as the trans Golgi network in hVSMCs, and trafficking from these compartments is required for FASL or FAS antibody-induced apoptosis [35]. Our data show that DISC formation induced by TIMP-3 expression is in intracellular vesicles that are rich in G_M1_-lipids as detected by co-localisation with CTxB, implying that FAS trafficking in G_M1_-lipid rich compartments is regulated by TIMP-3. Furthermore, the surface staining of FAS does not co-localise with CTxB, suggesting the surface clustering of FAS does not occur in G_M1_-lipid containing rafts (unpublished data), that has been observed in ligand and antibody-induced activation of FAS in type-I cells [36, 37], indicating surface clustering of FAS may not directly participate in FAS activation. We conclude that, similar to some other FASL-independent systems [25, 38, 39], TIMP-3-induced apoptosis of hVSMCs most likely occurs via intracellular activation of FAS.

c-FLIP is a caspase-8–like protein that can have an inhibitory effect on caspase-8 activation in DISC, but also promote apoptosis depending on cell type and expression level [40]. c-FLIP cellular localisation has been shown to be regulated by cell adhesion, which in turn regulates apoptosis [41]. Our data also indicate that TIMP-3 may induce apoptosis in hVSMCs in part through regulation of c-FLIP localisation, as well as increasing FAS cell surface clustering and regulating intracellular localisation, leading to DISC formation.

In conclusion, we have shown TIMP-3 activates apoptosis in hVSMCs via a FAS dependent type-I death receptor pathway that is independent of FASL and our findings are summarised in S7 Fig. Further studies are now needed to understand how TIMP-3 regulates the localisation of c-FLIP and if apoptosis is dependent on inhibition of additional metalloproteinases other than ADAM17, or if apoptosis is also partially independent of TIMP-3’s metalloproteinase inhibitory functions.

## Supporting information

S1 Fig RAdT3 infection of hVSMCs does not induce apoptosis via a type-II death receptor pathway. A. hVSMCs were infected for 48 h with either control adenovirus (RAd60) or adenovirus expressing Bcl2 (RAd-Bcl2) before the addition of vehicle control (DMSO) or 0.1 μM staurosporine for 16 h and cell lysates analysed for caspase-3 activity. Data are the mean ± SEM, n = 3. *** = p < 0.001, NS = Not Significant. B. hVSMCs were infected for 48 h with either RAd60 or RAdT3 before incubation with 1 μM mitotracker green and 0.5 μM TMRE. RAd60 infected cells were also incubated with 5 μM FCCP for 30 min prior to the addition of mitochondrial dyes as a positive control for mitochondrial depolarisation. Representative dot-plots of Mitotracker Green vs. TMRE fluorescence intensity after gating to remove cellular debris and doublets/aggregates of RAd60 infected cells (red), RAdT3 infected cells (green) and Rad60 infected cells incubated with FCCP (blue).
(TIF)

S2 Fig sFAS is decreased in conditioned medium from hVSMCs transduced with lentivirus expressing shRNA targeting FAS. hVSMCs were transduced with lentivirus conferring puromycin resistance alone (Puro), control non-targeting shRNA (Cont shRNA) or shRNA targeting FAS (FAS-1, -3, -4). Puromycin resistant cells were incubated in fresh medium for 72 h before soluble FAS (sFAS) was measured by ELISA in cell-conditioned medium. Data are the mean ± SEM, n = 3. *** = p < 0.001, NS = Not Significant.
(TIF)

S3 Fig FAS and FADD co-localise with Cholera toxin B-subunit conjugates. Human VSMCs were infected for 48 h with control (RAd60) or TIMP-3 expressing adenovirus (RAdT3). Cells were incubated with Cholera toxin B subunit (CTxB) AlexaFluor647 conjugates for 30 min in culture medium before fixing. Cells were stained with anti-FAS (IgM) and anti-FADD (IgG) followed with anti-IgM AlexaFluor488 and anti-goat AlexaFluor547 secondary antibodies and images captured by confocal microscopy. Colours for conjugates in the overlay image are CTxB; Blue, FAS; Green and FADD; Red.
(TIF)

S4 Fig Measurement of soluble FAS and caspase-3 activity in hVSMCs over-expressing TIMP-1, TIMP-2 or treated with MMP/ADAM inhibitors. hVSMCs were either infected with RAd60, RAd-T1 or RAd-T2 for 72 h before medium and cell lysates were harvested. Alternatively hVSMC were treated with either 0.1% v/v DMSO, 50 μM GM6001 or 10 μM BB-94 with medium and inhibitor changed every 24 before medium and cell lysates were harvested after 72 h. A. sFAS levels (pg ml^-1^) in cell conditioned medium were measured using a sFAS ELISA. Data are the mean ± SEM, n = 3. * = P < 0.05, NS = Not Significant. B. Caspase-3 activity was measured in cell lysates as described in the Experimental Procedures. Cell lysates from RAdT3 infected cells were used as a positive control. Data are the mean ± SEM, n = 3. *** = P < 0.001, NS = Not Significant.
(TIF)

S5 Fig Single cell image analysis of cell surface FAS clustering in hVSMCs. A. In order to demonstrate the monocolonal antibody CH-11 is able to measure cell surface FAS by high content image analysis hVSMCs were transduced with either lentivirus expressing non-targeting control shRNA (Cont shRNA) or virus expressing shRNA targeting FAS (FAS-4). Cells were seeded in Ibidi μ-well slides before incubation with either isotype control (IgM) or anti-FAS antibody CH-11 (FAS-Ab) followed by Alexfluor-488 anti IgM. Cells were then labelled with HCS CellMask^TM^ far-red dye and imaged using an iCys imaging cytometer. Data are the mean fluorescence level per cell ± SEM, n = 3. * = P < 0.05, ** = P < 0.01.
(TIF)

S6 Fig Single cell imaging analysis of cell surface FAS on hVSMC transduced with lentivirus targeting ADAM17. hVSMC were transduced with lentivirus expressing control non-targeting shRNA (shCont) or shRNA targeting ADAM17 (A17-3 and A17-4). Cell surface FAS was measured as described in the experimental procedures. **A.** Area of FAS cell surface spot-like structures per cell. **B.** Staining intensity within the FAS cell surface spot-like structures per cell. Data are the mean ± SEM, n = 3. No significance was detected between hVSMC transduced with control shRNA or shRNA targeting ADAM17.
(TIF)

S7 Fig Graphical summary. In the absence of TIMP-3, FAS is localised at the cell surface in small spot-like structure and intracellular vesicles where it co-localises with c-FLIP. ADAM17 is the predominant contributor to FAS shedding into the conditioned medium. High-levels of TIMP-3 expression lead to an increase in cellular FAS expression and an increase in FAS within the cell surface spots. This is accompanied by an increase in FAS within lipid-raft containing vesicles and is also associated with increased co-localisation with caspase-8 and FADD, detected in a complex by immunoprecipitation, indicating intracellular formation of DISC. The co-localisation of c-FLIP with FAS is lost and caspase-3 activation is associated with increased cleavage of PARP and nuclear fragmentation and cell death. Although TIMP-3 inhibits FAS shedding, depletion of ADAM17 alone cannot activate apoptosis, this observation, in combination with additional data, suggests increased TIMP-3 expression activates apoptosis partly via a proteinase independent mechanism.
(TIF)
