# Supplementary figures and images for: Tissue Inhibitor of Metalloproteinase–3 (TIMP-3) induces FAS dependent apoptosis in human vascular smooth muscle cells
Source: PLoS One. 2018 Apr 4;13(4):e0195116. doi: 10.1371/journal.pone.0195116 (PMC5884528; doi:10.1371/journal.pone.0195116)

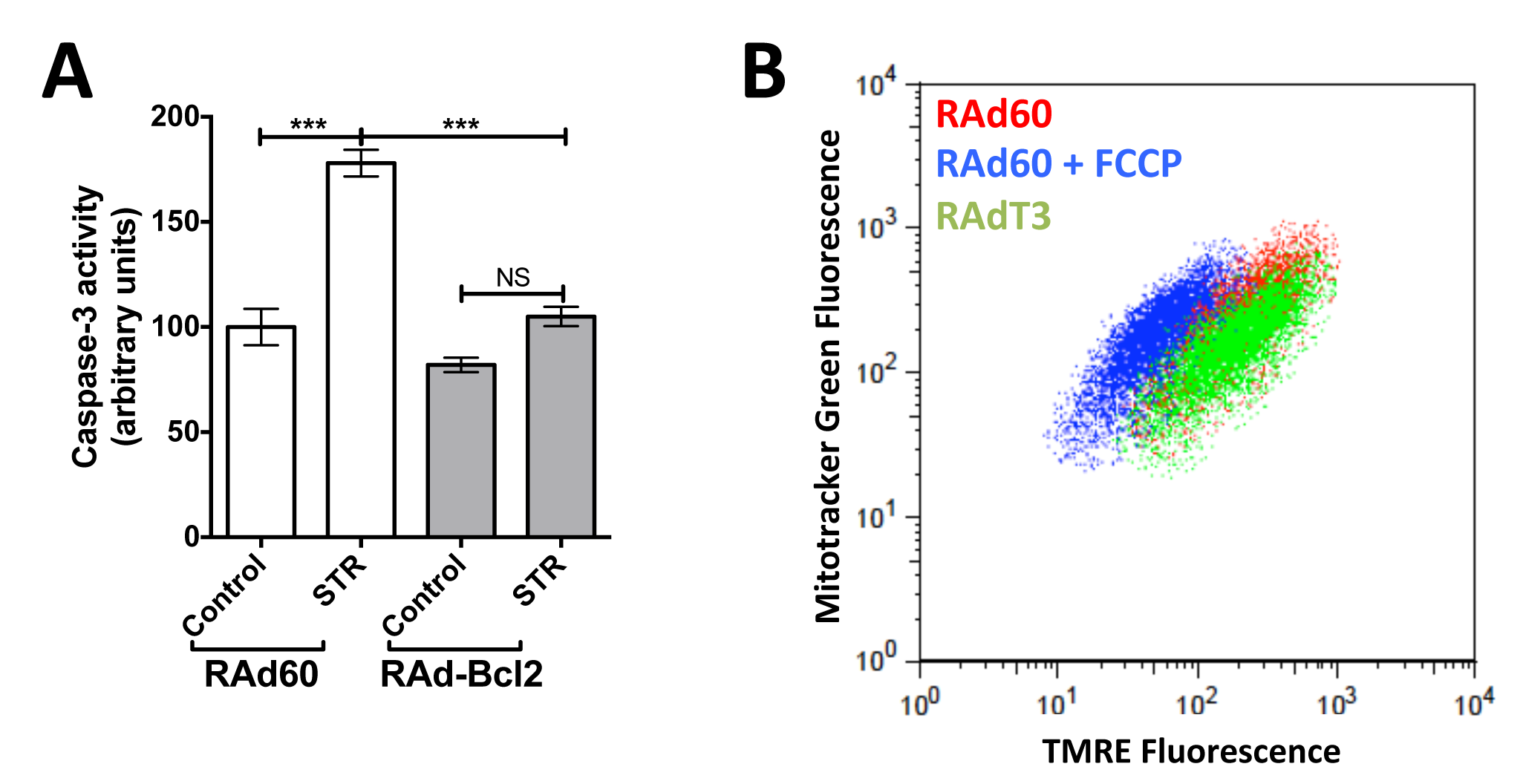

Supplement: S1 Fig — A. hVSMCs were infected for 48 h with either control adenovirus (RAd60) or adenovirus expressing Bcl2 (RAd-Bcl2) before the addition of vehicle control (DMSO) or 0.1 μM staurosporine for 16 h and cell lysates analysed for caspase-3 activity. Data are the mean ± SEM, n = 3. *** = p < 0.001, NS = Not Significant. B. hVSMCs were infected for 48 h with either RAd60 or RAdT3 before incubation with 1 μM mitotracker green and 0.5 μM TMRE. RAd60 infected cells were also incubated with 5 μM FCCP for 30 min prior to the addition of mitochondrial dyes as a positive control for mitochondrial depolarisation. Representative dot-plots of Mitotracker Green vs. TMRE fluorescence intensity after gating to remove cellular debris and doublets/aggregates of RAd60 infected cells (red), RAdT3 infected cells (green) and Rad60 infected cells incubated with FCCP (blue). (TIF) [file pone.0195116.s001.tif]

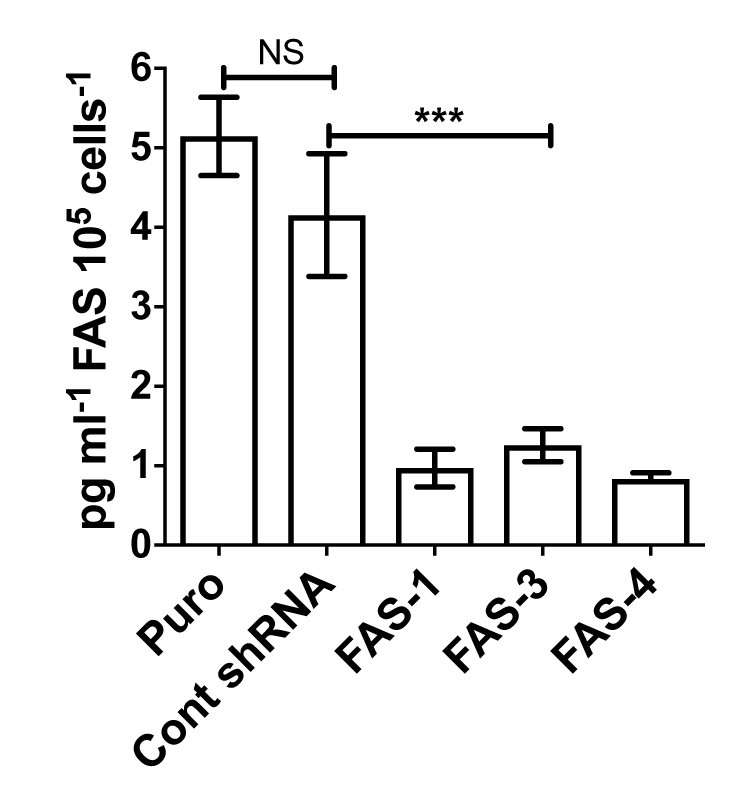

Supplement: S2 Fig — hVSMCs were transduced with lentivirus conferring puromycin resistance alone (Puro), control non-targeting shRNA (Cont shRNA) or shRNA targeting FAS (FAS-1, -3, -4). Puromycin resistant cells were incubated in fresh medium for 72 h before soluble FAS (sFAS) was measured by ELISA in cell-conditioned medium. Data are the mean ± SEM, n = 3. *** = p < 0.001, NS = Not Significant. (TIF) [file pone.0195116.s002.tif]

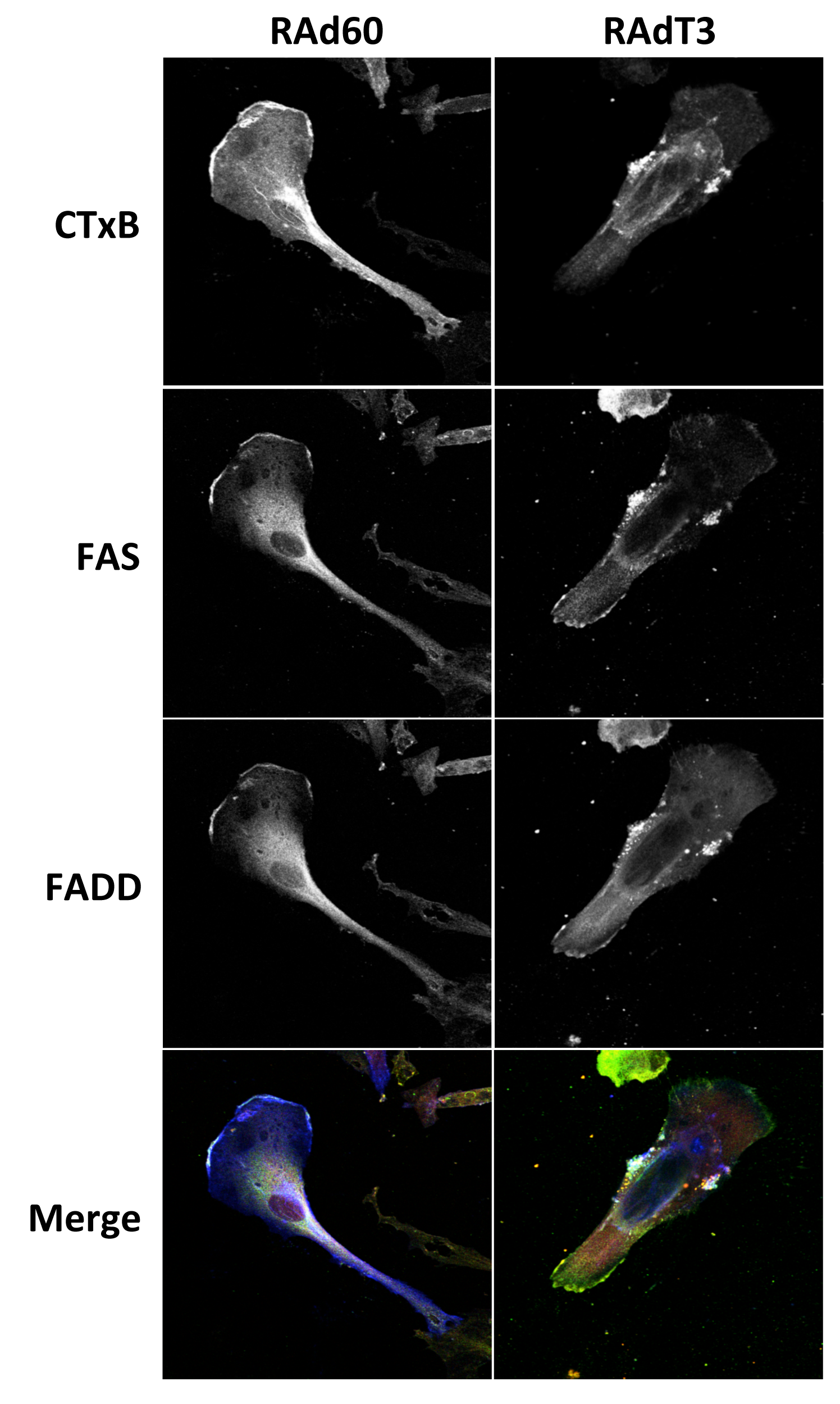

Supplement: S3 Fig — Human VSMCs were infected for 48 h with control (RAd60) or TIMP-3 expressing adenovirus (RAdT3). Cells were incubated with Cholera toxin B subunit (CTxB) AlexaFluor647 conjugates for 30 min in culture medium before fixing. Cells were stained with anti-FAS (IgM) and anti-FADD (IgG) followed with anti-IgM AlexaFluor488 and anti-goat AlexaFluor547 secondary antibodies and images captured by confocal microscopy. Colours for conjugates in the overlay image are CTxB; Blue, FAS; Green and FADD; Red. (TIF) [file pone.0195116.s003.tif]

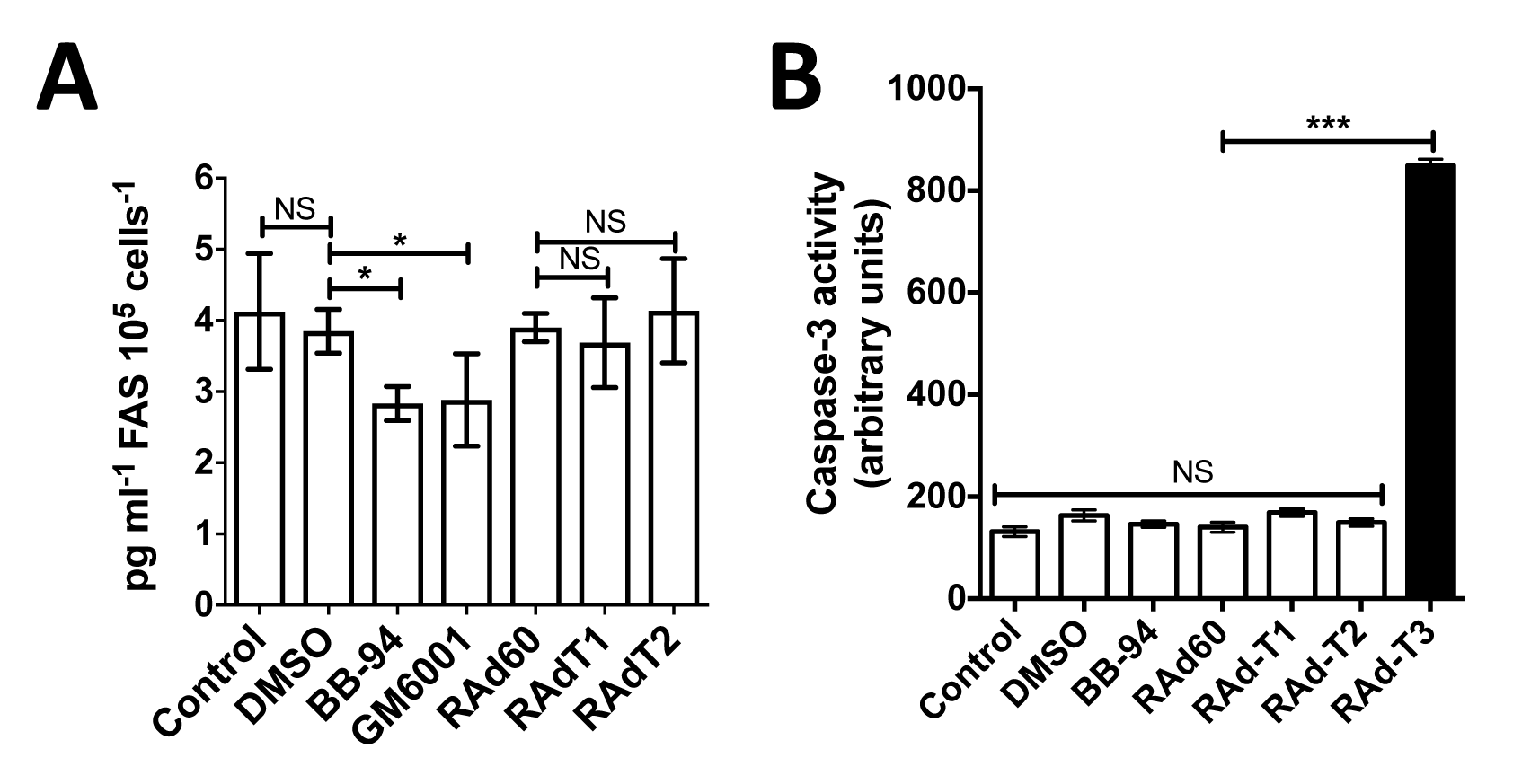

Supplement: S4 Fig — hVSMCs were either infected with RAd60, RAd-T1 or RAd-T2 for 72 h before medium and cell lysates were harvested. Alternatively hVSMC were treated with either 0.1% v/v DMSO, 50 μM GM6001 or 10 μM BB-94 with medium and inhibitor changed every 24 before medium and cell lysates were harvested after 72 h. A. sFAS levels (pg ml-1) in cell conditioned medium were measured using a sFAS ELISA. Data are the mean ± SEM, n = 3. * = P < 0.05, NS = Not Significant. B. Caspase-3 activity was measured in cell lysates as described in the Experimental Procedures. Cell lysates from RAdT3 infected cells were used as a positive control. Data are the mean ± SEM, n = 3. *** = P < 0.001, NS = Not Significant. (TIF) [file pone.0195116.s004.tif]

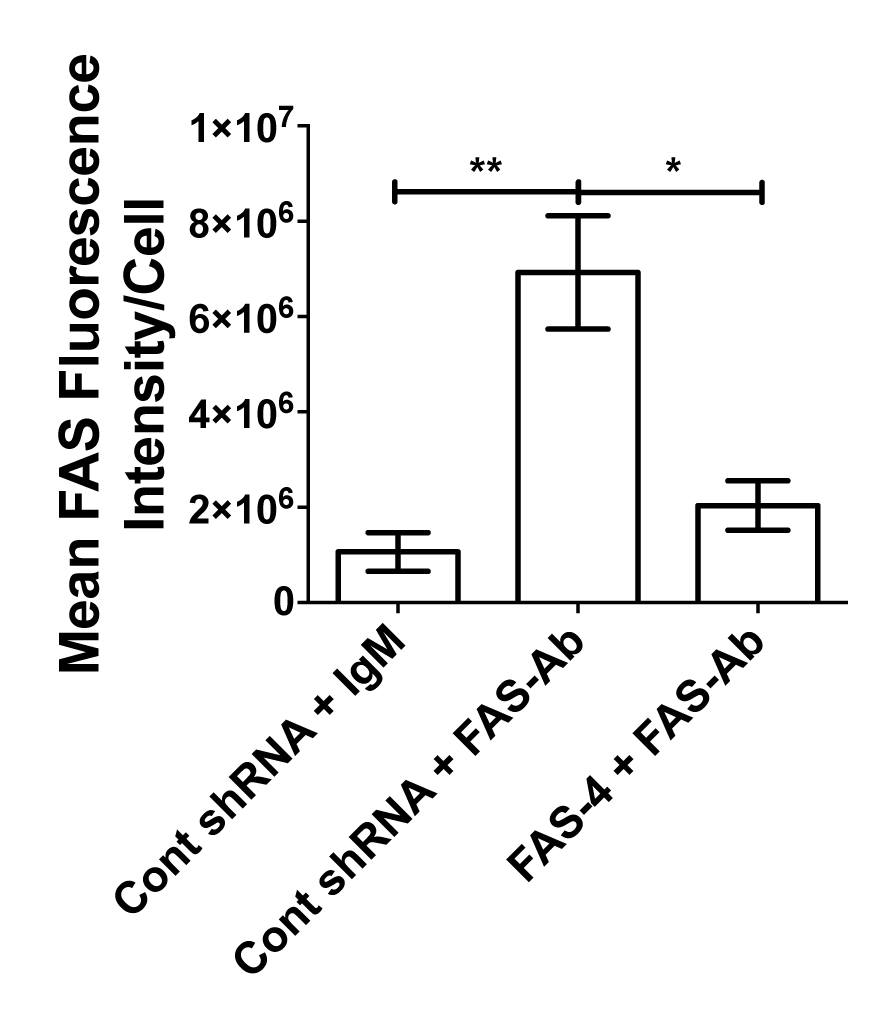

Supplement: S5 Fig — A. In order to demonstrate the monocolonal antibody CH-11 is able to measure cell surface FAS by high content image analysis hVSMCs were transduced with either lentivirus expressing non-targeting control shRNA (Cont shRNA) or virus expressing shRNA targeting FAS (FAS-4). Cells were seeded in Ibidi μ-well slides before incubation with either isotype control (IgM) or anti-FAS antibody CH-11 (FAS-Ab) followed by Alexfluor-488 anti IgM. Cells were then labelled with HCS CellMaskTM far-red dye and imaged using an iCys imaging cytometer. Data are the mean fluorescence level per cell ± SEM, n = 3. * = P < 0.05, ** = P < 0.01. (TIF) [file pone.0195116.s005.tif]

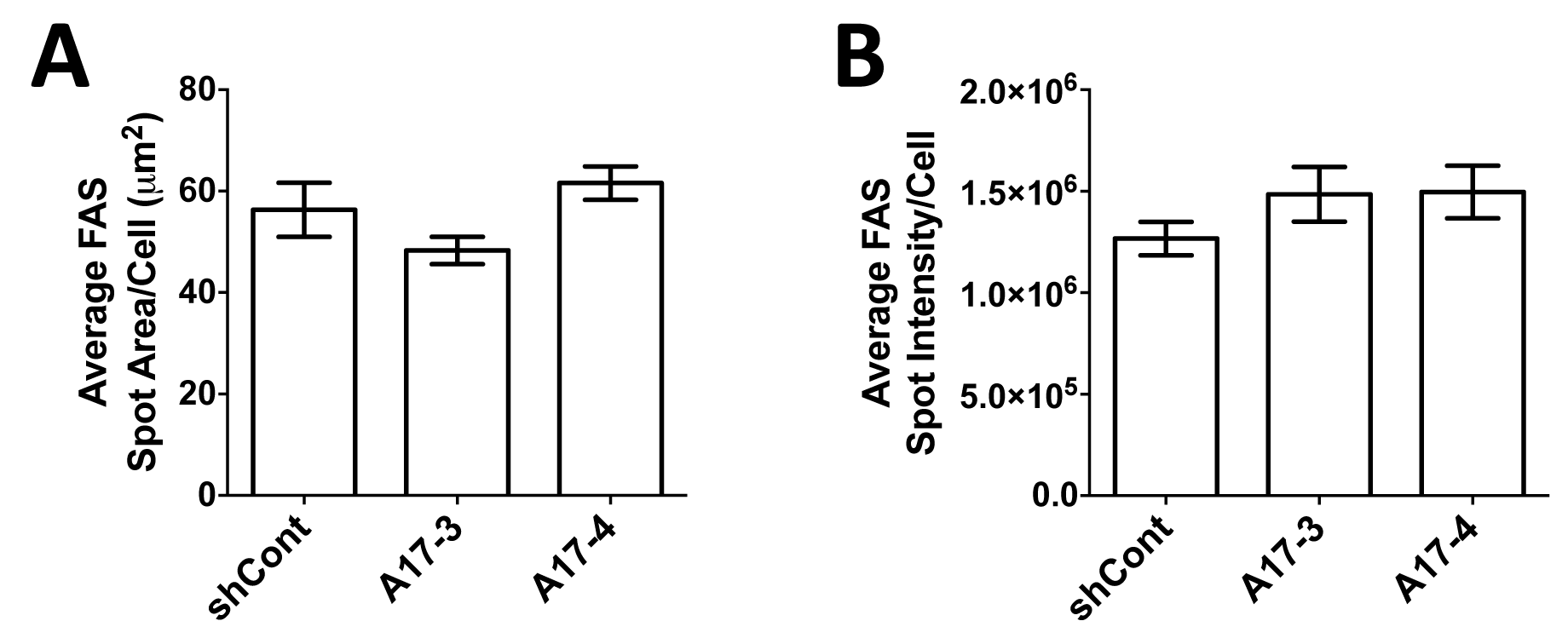

Supplement: S6 Fig — hVSMC were transduced with lentivirus expressing control non-targeting shRNA (shCont) or shRNA targeting ADAM17 (A17-3 and A17-4). Cell surface FAS was measured as described in the experimental procedures. A. Area of FAS cell surface spot-like structures per cell. B. Staining intensity within the FAS cell surface spot-like structures per cell. Data are the mean ± SEM, n = 3. No significance was detected between hVSMC transduced with control shRNA or shRNA targeting ADAM17. (TIF) [file pone.0195116.s006.tif]

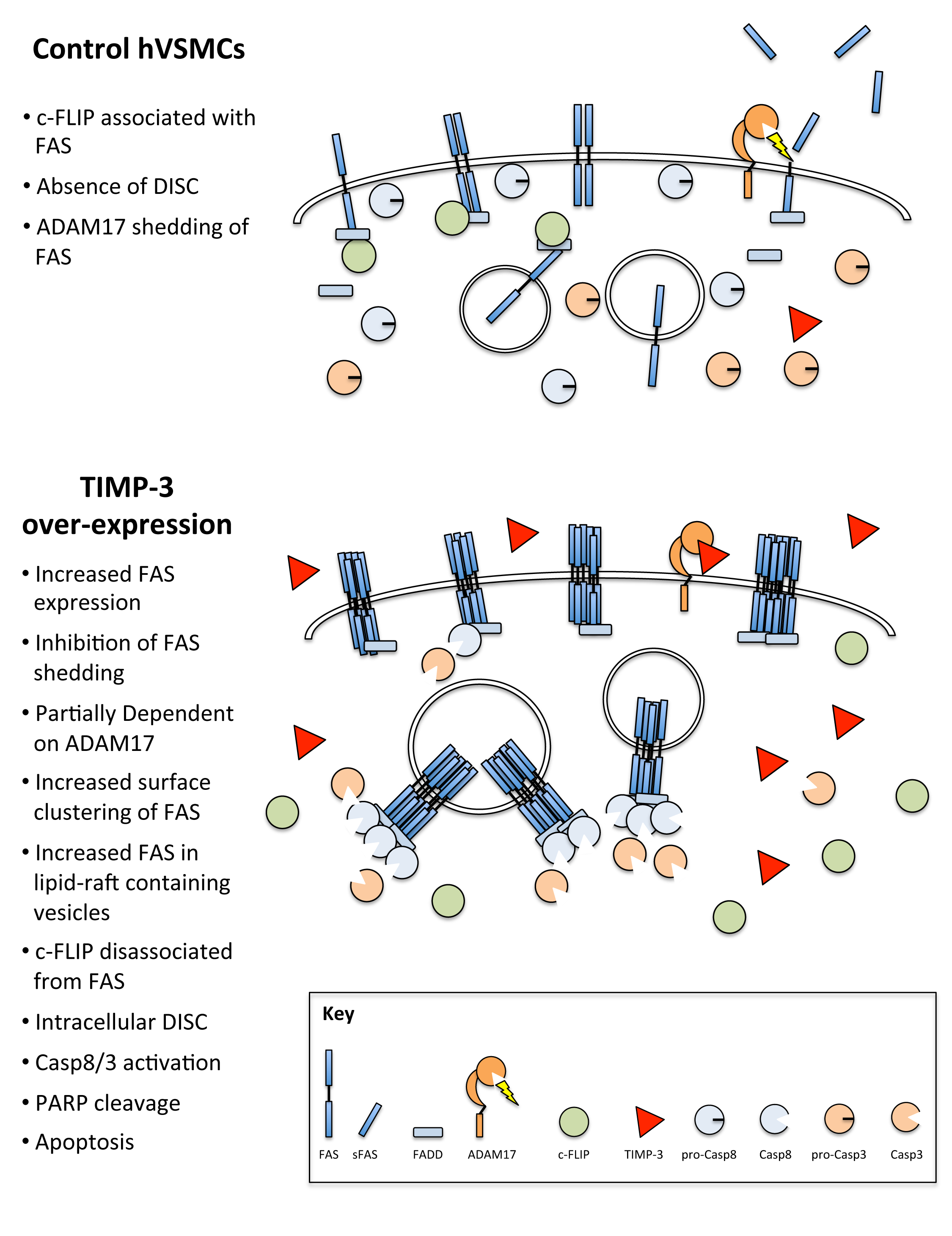

Supplement: S7 Fig — In the absence of TIMP-3, FAS is localised at the cell surface in small spot-like structure and intracellular vesicles where it co-localises with c-FLIP. ADAM17 is the predominant contributor to FAS shedding into the conditioned medium. High-levels of TIMP-3 expression lead to an increase in cellular FAS expression and an increase in FAS within the cell surface spots. This is accompanied by an increase in FAS within lipid-raft containing vesicles and is also associated with increased co-localisation with caspase-8 and FADD, detected in a complex by immunoprecipitation, indicating intracellular formation of DISC. The co-localisation of c-FLIP with FAS is lost and caspase-3 activation is associated with increased cleavage of PARP and nuclear fragmentation and cell death. Although TIMP-3 inhibits FAS shedding, depletion of ADAM17 alone cannot activate apoptosis, this observation, in combination with additional data, suggests increased TIMP-3 expression activates apoptosis partly via a proteinase independent mechanism. (TIF) [file pone.0195116.s007.tif]
